# The Genetic and Neural Substrates of Externalizing Behavior

**DOI:** 10.1016/j.bpsgos.2021.09.007

**Published:** 2021-10-06

**Authors:** Bart Baselmans, Anke R. Hammerschlag, Stephany Noordijk, Hill Ip, Matthijs van der Zee, Eco de Geus, Abdel Abdellaoui, Jorien L. Treur, Dennis van ’t Ent

**Affiliations:** a Institute for Molecular Bioscience, The University of Queensland, Brisbane, Queensland, Australia; b Child Health Research Centre, The University of Queensland, Brisbane, Queensland, Australia; c Department of Biological Psychology, Vrije Universiteit Amsterdam, Amsterdam, the Netherlands; d Amsterdam Public Health research institute, Amsterdam University Medical Centre, Amsterdam, the Netherlands; e Department of Psychiatry, Amsterdam UMC, University of Amsterdam, Amsterdam, the Netherlands

**Keywords:** Disruptive behavior, Externalizing behavior, Gene set analysis, Genetic correlation, GWAS, Mendelian randomization, N-GWAMA, Risk-taking behavior, Stratified LD-score regression

## Abstract

**Background:**

To gain more insight into the biological factors that mediate vulnerability to display externalizing behaviors, we leveraged genome-wide association study summary statistics on 13 externalizing phenotypes.

**Methods:**

After data classification based on genetic resemblance, we performed multivariate genome-wide association meta-analyses and conducted extensive bioinformatic analyses, including genetic correlation assessment with other traits, Mendelian randomization, and gene set and gene expression analyses.

**Results:**

The genetic data could be categorized into disruptive behavior (DB) and risk-taking behavior (RTB) factors, and subsequent genome-wide association meta-analyses provided association statistics for DB and RTB (*N*_eff_ = 523,150 and 1,506,537, respectively), yielding 50 and 257 independent genetic signals. The statistics of DB, much more than RTB, signaled genetic predisposition to adverse cognitive, mental health, and personality outcomes. We found evidence for bidirectional causal influences between DB and substance use behaviors. Gene set analyses implicated contributions of neuronal cell development (DB/RTB) and synapse formation and transcription (RTB) mechanisms. Gene-brain mapping confirmed involvement of the amygdala and hypothalamus and highlighted other candidate regions (cerebellar dentate, cuneiform nucleus, claustrum, paracentral cortex). At the cell-type level, we noted enrichment of glutamatergic neurons for DB and RTB.

**Conclusions:**

This bottom-up, data-driven study provides new insights into the genetic signals of externalizing behaviors and indicates that commonalities in genetic architecture contribute to the frequent co-occurrence of different DBs and different RTBs, respectively. Bioinformatic analyses supported the DB versus RTB categorization and indicated relevant biological mechanisms. Generally similar gene-brain mappings indicate that neuroanatomical differences, if any, escaped the resolution of our methods.

Externalizing psychopathologies are highly common in the general population (1, 2, 3, 4), and expressions of externalizing behavior including bullying, violence, delinquent activities, risk taking, and other related actions have a negative impact on individual prospects, local communities, and our society as a whole (5). It is therefore vitally important to gain more insight into the underlying (neuro)biological processes to understand why some individuals are more susceptible to displaying externalizing behaviors than others.

Externalizing symptoms generally co-occur substantially (6), which suggests commonalities in their etiologies (3,7). This is supported by indications for common genetic and environmental risk factors based on twin family studies (8, 9, 10, 11) and evidence for shared brain substrates from neurobiological studies (12, 13, 14). There is also evidence for causal relationships, particularly between substance use and (other) externalizing symptoms (15), a topic of high relevance for public health.

To broaden our understanding of common genetic and neurobiological backgrounds that underlie the co-occurrence of different externalizing behaviors, we leveraged publicly available genome-wide association study (GWAS) summary statistics on lifetime cannabis use (16), antisocial behavior (17), aggressive behavior (18), four item indicators of angriness and irritability from UK Biobank (19), and attention-deficit/hyperactivity disorder (ADHD) diagnosis (20). Furthermore, we added five items from a recent study on the genetics of risk tolerance (21). The 13 included datasets are summarized in Table 1.

**Table 1:** GWAS Summary Statistics Included in the Multivariate Genome-wide Association Meta-analysis

Phenotype	Sample Size	Source
Aggression	18,988	Pappa *et al*. (18)
Angry Outbursts	71,196	Sudlow *et al*. (19)
Extreme Irritable	157,357	Sudlow *et al*. (19)
Irritability	501,652	Sudlow *et al*. (19)
Irritable for 2 Days	202,883	Sudlow *et al*. (19)
ADHD	53,293	Demontis *et al*. (20)
Antisocial Behavior	25,781	Tielbeek *et al*. (17)
General Risk Tolerance	466,571	Karlsson Linnér *et al*. (21)
Drinks per Week	414,343	Karlsson Linnér *et al*. (21)
Ever Smoker	518,633	Karlsson Linnér *et al*. (21)
Number of Sexual Partners	370,711	Karlsson Linnér *et al*. (21)
Automobile Speeding Propensity	404,291	Karlsson Linnér *et al*. (21)
Lifetime Cannabis Use	184,765	Pasman *et al*. (16)

ADHD, attention-deficit/hyperactivity disorder; GWAS, genome-wide association study.

Using these data, we first identified clusters of traits with high genetic resemblance that together comprise the higher-order externalizing behavior dimension. Subsequently, the GWAS data of the clusters were meta-analyzed using N-weighted meta-analysis (22). To characterize the genetic information in the meta-analyzed data, we evaluated the genetic relationships of the clusters with other phenotypes. In addition, for an identified cluster characterized by disruptive-type behaviors, we tested for causal relationship with smoking and alcohol consumption using bidirectional Mendelian randomization (MR). Previous studies have not been able to uncover the causal nature and direction of the relationship between disruptive behaviors (DBs) and smoking and alcohol use, two major risk factors for morbidity and mortality. Finally, biological annotation analyses were performed to identify associated brain regions and cell types.

## Methods and Materials

### Phenotype Selection

Based on the disinhibited externalizing spectrum described by Krueger *et al.* (1), we collected GWAS summary statistics for 13 externalizing phenotypes (Table 1). Only samples with a European/North-American ancestry (23) were included (see Supplement 1).

### Identification of Genetic Factor Structures

To identify genetic factor structures among the 13 included phenotypes, we first applied linkage disequilibrium score regression (LDSC) (24,25) to compute pairwise genetic correlations (*r*_*g*_) (see Supplement 1).

Second, knowing the genetic correlations, we examined relationships using hierarchical clustering with (1 − *r*_*g*_) as genetic distance measures between phenotypes and with linkage based on Ward’s method (26). We followed the Calinski-Harabasz criterion to indicate the optimal number of clusters (27).

Third, in addition to hierarchical clustering, we applied factor analysis on the GWAS summary statistics using genomic structural equation modeling (28) (see Supplement 1).

### Multivariate N-Weighted Genome-wide Association Meta-analysis

Univariate GWAS summary statistics were separately combined for identified factors using N-weighted genome-wide association meta-analysis (GWAMA) (22), which is robust against sample overlap (see Supplement 1).

### Genetic Relationships With Other Traits

We computed pairwise genetic correlations using LDSC (24,25) for our identified factors with 61 additional phenotypes in the following categories: mental health, cognition and socioeconomic status, personality, social, substance use, cardiovascular disease risk, physical health, anthropomorphic, and reproduction (see Table S6 in Supplement 2 and Supplement 1 for details).

### Mendelian Randomization

For one of the identified clusters characterized by DBs, we applied MR to test for causal relationship with substance use behaviors (see Supplement 1).

### Gene Associations

We performed gene-based analyses in MAGMA version 1.08 (http://ctg.cncr.nl/software/magma) (29) with the N-weighted GWAMA summary statistics of the identified factors as input. The gene test statistics are defined as the mean single nucleotide polymorphism (SNP) association using the sum of −log (SNP *p* value).

### Tissue-Type Associations

We used the gene associations of the two factors as input for a tissue-type analysis using MAGMA version 1.08 (29). We investigated tissue-specific gene expression values as gene properties using 53 tissues from the Genotype-Tissue Expression project v.7 (30) (see Supplement 1).

### Gene Set Associations

We continued with competitive gene set analyses using MAGMA version 1.08 (29) to test whether the genes in a gene set are more strongly associated with the factor phenotypes than the other genes in the genome (see Supplement 1).

### Conditional Analyses for Tissue Types and Gene Sets

For the significantly associated tissue types and gene sets, we performed conditional analyses using MAGMA version 1.08 (29) to evaluate redundancy between associations (see Supplement 1).

### Stratified LDSC of Tissue Types

We applied stratified LDSC to investigate which tissues and cell types are enriched for the identified factors (see Supplement 1).

### Stratified LDSC of Local Gene Expression Across the Human Brain

To identify brain regions where genes relevant for externalizing behavior factors are differently expressed, we computed stratified LD scores based on differential gene expression, using data from 3707 gene expression measurements across 211 different brain regions (31) (see Supplement 1).

### Stratified LDSC of Brain Cell Types

We obtained a matrix of gene counts for single nuclei (*n* = 14,963) from the prefrontal cortex and hippocampus of multiple human donors studied by Habib *et al.* (32). We subsequently determined the differential expression of genes in seven types of brain cells: GABAergic (gamma-aminobutyric acidergic) interneurons, excitatory neurons, astrocytes, oligodendrocytes, microglia, endothelial cells, and neural stem cells (32) (see Supplement 1).

## Results

### Identification of Genetic Factor Structure

**Figure 1:**
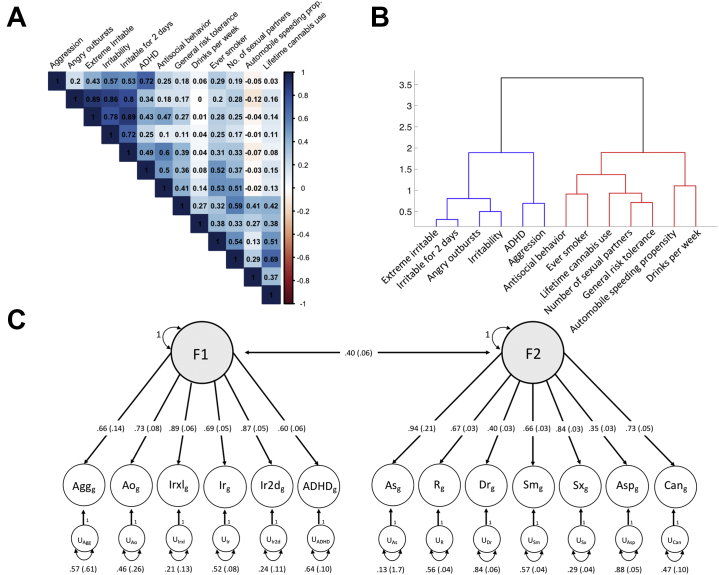
Genetic factor structure of externalizing behaviors. **(A)** Genetic correlations between the externalizing phenotypes calculated by linkage disequilibrium score regression. **(B)** Hierarchical clustering dendrogram based on genetic resemblance of the externalizing phenotypes. Blue represents the disruptive behavior cluster, and red represents the risk-taking behavior cluster. **(C)** Based on the results of an exploratory factor analysis of the genetic correlations presented in panel **(A)**, a confirmatory factor model with two correlated genetic factors was specified using genomic structural equation modeling. In this model, the common factors account for the genetic covariation among the externalizing traits, i.e., each of the two common genetic factors represents variation in genetic liability that is shared across the phenotypes that load on it. Disruptive behavior represents shared genetic liability among disorders characterized by disruptive behavior, and risk-taking behavior represents the shared liability for risk-taking behavior. One-headed arrows connecting the common genetic factors to the individual traits represent standard loadings, which can be interpreted as coefficients from a regression of the true genetic liability for the trait on the common factor. Two-headed arrows connecting the genetic components represent their correlations. Two-headed arrows connecting the genetic components of the individual traits to themselves represent residual genetic variances and correspond to the proportion of heritable variation in liability to each individual trait that is unexplained by the two factors. ADHD, attention-deficit/hyperactivity disorder; Agg, aggression; Ao, angry outbursts; As, antisocial behavior; Asp, automobile speeding propensity; Can, lifetime cannabis use; Dr, drinks per week; Irxl, extreme irritable; Ir, irritability; Ir2d, irritable for 2 days; R, general risk tolerance; Sm, ever smoker; Sx, number of sexual partners.

LDSC (Figure 1A) indicated substantial genetic correlations, particularly among phenotypes characterized by disruptive-type behaviors (e.g., aggression and ADHD: *r*_*g*_ = 0.72, SE = 0.18) and phenotypes characterized by risk-taking behaviors (RTBs) (e.g., cannabis use and number of sexual partners: *r*_*g*_ = 0.69, SE = 0.02), with less genetic overlap between pairs of phenotypes characterized by the different behavior types (e.g., aggression and cannabis use: *r*_*g*_ = 0.03, SE = 0.13). Hierarchical clustering on genetic resemblance (Figure 1B) confirmed this categorization into DBs (aggression, angry outbursts, different measures of irritability, and ADHD) and RTBs (antisocial behavior, general risk tolerance, drinks per week, ever smoker, number of sexual partners, automobile speeding propensity, and lifetime cannabis use). Exploratory factor analysis by genomic structural equation modeling supported a division into two phenotypic clusters by demonstrating a substantial increase in explained variance from 34% for a one-factor model to 53% for a two-factor model, but a much more moderate increase to 58% for a three-factor model (Figure 1C; Tables S1–S3 in Supplement 2). The two-factor exploratory factor analysis model assigned similar phenotypes to each factor as our hierarchical clustering approach. The loadings of the two-factor model, however, did not support a clear allocation for ADHD and antisocial behavior (Table S1 in Supplement 2). For these phenotypes, we followed the hierarchical clustering results, assigning ADHD to factor 1 (DB) and antisocial behavior to factor 2 (RTB).

### Multivariate GWAMA

**Figure 2:**
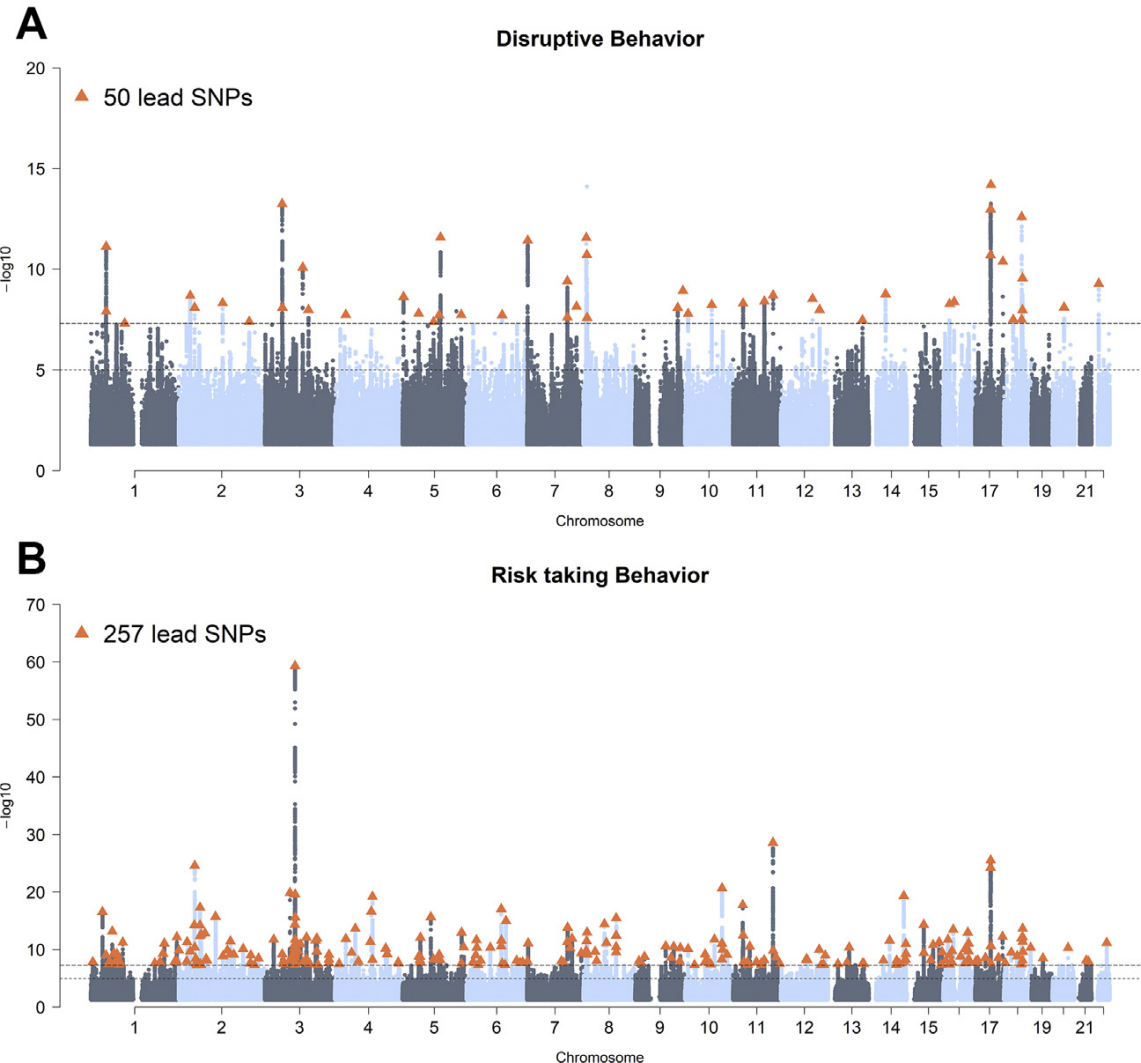
Manhattan plots of the meta-analyzed phenotype genome-wide association study data. **(A)** Disruptive behavior factor. **(B)** Risk-taking behavior factor. The x-axis represents the chromosomal position, and the y-axis represents the significance on a −log_10_ scale. Each approximately independent genome-wide significant association (lead SNP) is marked by a triangle (*p* < 5 × 10^−8^). SNP, single nucleotide polymorphism.

For both factors, we subsequently meta-analyzed the phenotype GWAS data using N-weighted GWAMA. For the DB factor, we identified 50 independent genome-wide significant SNPs at 42 loci (*N*_eff_ = 523,150) (Figure 2A; Table S4 in Supplement 2). For RTB, we identified 257 independent genome-wide significant SNPs at 194 genomic risk loci (*N*_eff_ = 1,506,537) (Figure 2B; Table S5 in Supplement 2). The LD score intercepts were close to 1 for both DB (intercept = 1.0167, SE = 0.0086; LDSC ratio = 0.0367, SE = 0.0156) and RTB (intercept = 1.0031, SE = 0.0132; LDSC ratio = 0.0242, SE = 0.0106), indicating that neither population stratification nor sample overlap, but rather an increase of polygenic signal, was driving the SNP associations. The SNP heritability as defined by LDSC was 0.0396 (SE = 0.0015) for DB and 0.022 (SE = 0.0007) for RTB. The genetic correlation between DB and RTB was 0.33 (SE = 0.02).

### Genetic Relationships With Other Traits

**Figure 3:**
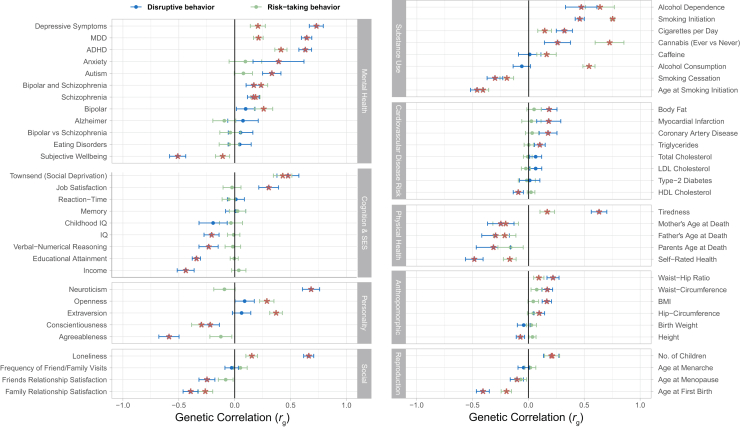
Genetic correlations with other phenotypes for disruptive behavior (blue) and risk-taking behavior (green) factors. Points represent the correlation estimates, and lines represent the 95% confidence intervals. Significant associations, after correction for multiple testing, are marked by orange stars. ADHD, attention-deficit/hyperactivity disorder; BMI, body mass index; HDL, high-density lipoprotein; LDL, low-density lipoprotein; MDD, major depressive disorder; SES, socioeconomic status.

Figure 3 and Table S7 in Supplement 2 show pairwise genetic correlations for DB and RTB with 61 additional phenotypes. DB, much more than RTB, showed a pattern of genetic overlap pointing to adverse outcomes of cognition, socioeconomic status, several mental and physical health measures, and personality. For example, DB showed a negative genetic correlation with educational attainment (*r*_*g*_ = −0.34, SE = 0.02) and income (*r*_*g*_ = −0.44, SE = 0.04), whereas genetic correlations for RTB with these phenotypes were close to zero. In addition, DB was more positively correlated with depressive symptoms (*r*_*g*_ = 0.73, SE = 0.03) and neuroticism (*r*_*g*_ = 0.68, SE = 0.04) and more negatively correlated with agreeableness (*r*_*g*_ = −0.59, SE = 0.05) compared with RTB (depressive symptoms: *r*_*g*_ = 0.21, SE = 0.03; neuroticism: *r*_*g*_ = −0.09, SE = 0.005; agreeableness: *r*_*g*_ = −0.12, SE = 0.05).

### Causal Relationship of DB With Smoking and Alcohol Use

We tested for bidirectional causal effects between DB (which does not include substance use phenotypes) and measures of smoking (33) and alcohol use (34) using MR. We focused on these specific relationships because DBs and substance use are particularly strongly associated, and knowledge of (the direction of) potential causal effects has major public health implications. When DB was the exposure variable, inverse variance weighted analyses provided strong evidence for causal effects such that DB increases the odds of smoking initiation (β = 0.39, 95% CI = 0.24–0.54, *p* = 2.0 × 10^−7^) and decreases the odds of being able to successfully quit smoking (β = −0.20, 95% CI = −0.33 to −0.07, *p* = .002) (Table 2; Figure S1 in Supplement 1). Effect sizes and statistical evidence for a causal relationship between DB and smoking initiation were broadly consistent across the different MR methods. However, for DB to smoking cessation, the weighted mode and MR-Egger did not support a causal relationship (Table 2). Some evidence for pleiotropic effects was provided by a significant Cochran’s heterogeneity test (Cochran’s Q *p* value = 2.4 × 10^−33^) (Table S8 in Supplement 2) but not supported by the MR-Egger intercept (only DB → smoking initiation) (Table S9 in Supplement 2). Steiger filtering and MR-PRESSO did not affect the results and continued to support causal effects (Tables S10–S12 in Supplement 2). No consistent results were found for a causal relationship between DB and number of cigarettes smoked per day (Table 2; Table S9 in Supplement 2).

**Table 2:** Results of the Two-Sample Bidirectional Mendelian Randomization Analyses Between DB and Smoking and Alcohol Use Behavior

Exposure	Outcome	SNPs, *n*	*F*	IVW			Weighted Median			Weighted Mode			MR-Egger (SIMEX)			GSMR			
				β	95% CI	*p*	β	95% CI	*p*	β	95% CI	*p*	β	95% CI	*p*	SNPs, *n*	β	95% CI	*p*
DB	Smoking initiation	40	37.41	0.39	0.24 to 0.54	2.0 × 10^−7^	0.25	0.13 to 0.37	3.2 × 10^−5^	0.15	−0.06 to 0.36	.180	0.43	0.09 to 0.77	.017	33	0.342	0.27 to 0.41	1.7 × 10^−20^
DB	Cigarettes/day	38	37.61	0.14	0.01 to 0.27	.029	−0.01	−0.12 to 0.10	.895	−0.06	−0.16 to 0.04	.284	−0.31	−0.60 to −0.02	.041	34	0.178	0.09 to 0.26	3.3 × 10^−5^
DB	Smoking cessation	40	37.33	−0.20	−0.33 to −0.07	.002	−0.11	−0.25 to 0.03	.118	0.02	−0.24 to 0.28	.873	0.17	−0.11 to 0.45	.254	37	−0.190	−0.29 to −0.09	9.9 × 10^−5^
DB	Alcohol/week	40	37.41	−0.01	−0.04 to 0.02	.472	−0.002	−0.03 to 0.03	.897	0.01	−0.02 to 0.04	.545	−0.04	−0.11 to 0.03	.341	35	−0.020	−0.04 to 0.00	.043
DB	Alcohol use disorder	46	37.00	0.26	0.08 to 0.44	.004	0.16	−0.10 to 0.42	.245	0.14	−0.12 to 0.40	.288	n.a.	n.a.	n.a.	43	0.310	0.10 to 0.52	.003
Smoking Initiation	DB	317	27.53	0.17	0.14 to 0.20	9.9 × 10^−31^	0.15	0.12 to 0.18	8.0 × 10^−21^	0.15	0.05 to 0.25	.004	n.a.	n.a.	n.a.	296	0.186	0.17 to 0.21	6.4 × 10^−72^
Alcohol/week	DB	80	28.61	0.04	−0.13 to 0.21	.632	0.052	−0.12 to 0.22	.543	−0.17	−2.07 to 1.73	.859	−0.24	−0.54 to 0.06	.133	74	0.0719	−0.05 to 0.20	.261
Alcohol Dis. 5 × 10^−8^	DB	7	26.35	−0.02	−0.06 to 0.02	.299	−0.02	−0.06 to 0.02	.480	−0.01	−0.08 to 0.06	.833	n.a.	n.a.	n.a.	7	0.00016	−0.04 to 0.04	.993
Alcohol Dis. 1 × 10^−5^	DB	25	21.94	−0.01	−0.03 to 0.01	.328	−0.01	−0.03 to 0.01	.654	−0.01	−0.06 to 0.04	.652	n.a.	n.a.	n.a.	24	−0.0078	−0.02 to 0.01	.365

n.a. indicates that MR-Egger results were not reported because of limited reliability based on the *I*^*2*^ measure being <0.60. *F* > 10 generally indicates the instrument is sufficiently strong.

DB, disruptive behavior; Dis., disorder; F, F-statistic indicating instrument strength; GSMR, generalized summary data–based Mendelian randomization; IVW, inverse variance weighted regression; SIMEX, simulation extrapolation; SNP, single nucleotide polymorphism.

From DB to alcohol use disorder, there was evidence for a causal increasing effect (inverse variance weighted analysis: β = 0.26, 95% CI = 0.08–0.44, *p* = .004), which was consistent (but weaker) in direction of effect across multiple MR methods (Table 2). MR-Egger was not available owing to low reliability; however, there was no heterogeneity based on Cochran’s Q (*p* = .457) (Table S8 in Supplement 2). Steiger filtering and MR-PRESSO did not change the outcome (Tables S10–S12 in Supplement 2).

In the opposite direction, we found strong evidence for a causal, increasing effect of smoking initiation on DB (inverse variance weighted analysis: β = 0.17, 95% CI = 0.14–0.20, *p* = 9.9 × 10^−31^), which was consistent across weighted median, weighted mode, and generalized summary data–based MR (MR-Egger not available). Steiger filtered analyses and MR-PRESSO did not change the results (Table 2; Tables S10–S12 in Supplement 2). However, there was marked evidence for heterogeneity (Cochran’s Q *p* = 7.3 × 10^−46^) (Table S8 in Supplement 2). There was no clear evidence for causal effects of alcohol use on DB.

### Biological Annotations

#### Gene Associations

Genome-wide gene-based association analysis identified 81 genes significantly associated with DB (Table S13 in Supplement 2) and 318 genes significantly associated with RTB (Table S14 in Supplement 2) after Bonferroni correction. *CADM2* showed an exceptionally strong association (2.10 × 10^−29^) with RTB.

#### Tissue-Type Associations

We used the gene-based test statistics as input for gene set analysis in MAGMA. Starting with a tissue-type analysis, we found significant associations at the Bonferroni level for nine brain regions for DB and 13 brain regions for RTB (Tables S15 and S16 in Supplement 2). Conditional analyses indicated that only the frontal cortex showed an independent association with DB, while independent associations were limited to the frontal cortex and cerebellum for RTB.

#### Gene Set Associations

Subsequent gene set analyses identified three gene sets for DB involved in cell development and 13 gene sets for RTB involved in cell development, synapse formation, and transcription (Tables S17 and S18 in Supplement 2). Conditional gene set analyses on the three gene sets associated with DB showed that the three gene set associations were highly related (Table S17 in Supplement 2). In addition, the conditional analyses of the 13 gene sets identified for RTB traits revealed redundancy for multiple gene sets related to synapse and cell development and transcription, leaving seven gene sets with independent association (Table S18 in Supplement 2).

#### Stratified LDSC

Complementary to gene set analysis, we applied stratified LDSC to study effects on 10 general tissue-type groups. Consistent with the gene set analyses, these analyses indicated significant enrichment after Bonferroni correction of the central nervous system (CNS) for both DB (Z_CNS_ = 6.84) (Table S19 in Supplement 2) and RTB (Z_CNS_ = 7.97) (Table S20 in Supplement 2). It has to be noted that other tissues were associated with DB and RTB as well (e.g., liver, kidney), but these associations were considerably weaker (Tables S19 and S20 in Supplement 2). Zooming in on associations on the cell-type level for DB revealed 45 significant cell type–specific annotations after Bonferroni correction, of which the top 15 all involved the CNS (Table S21 in Supplement 2). A similar pattern was observed for RTB, where we found 96 significant cell type–specific annotations, of which 32 indicated CNS involvement (Table S22 in Supplement 2).

#### Differential Gene Expression

**Figure 4:**
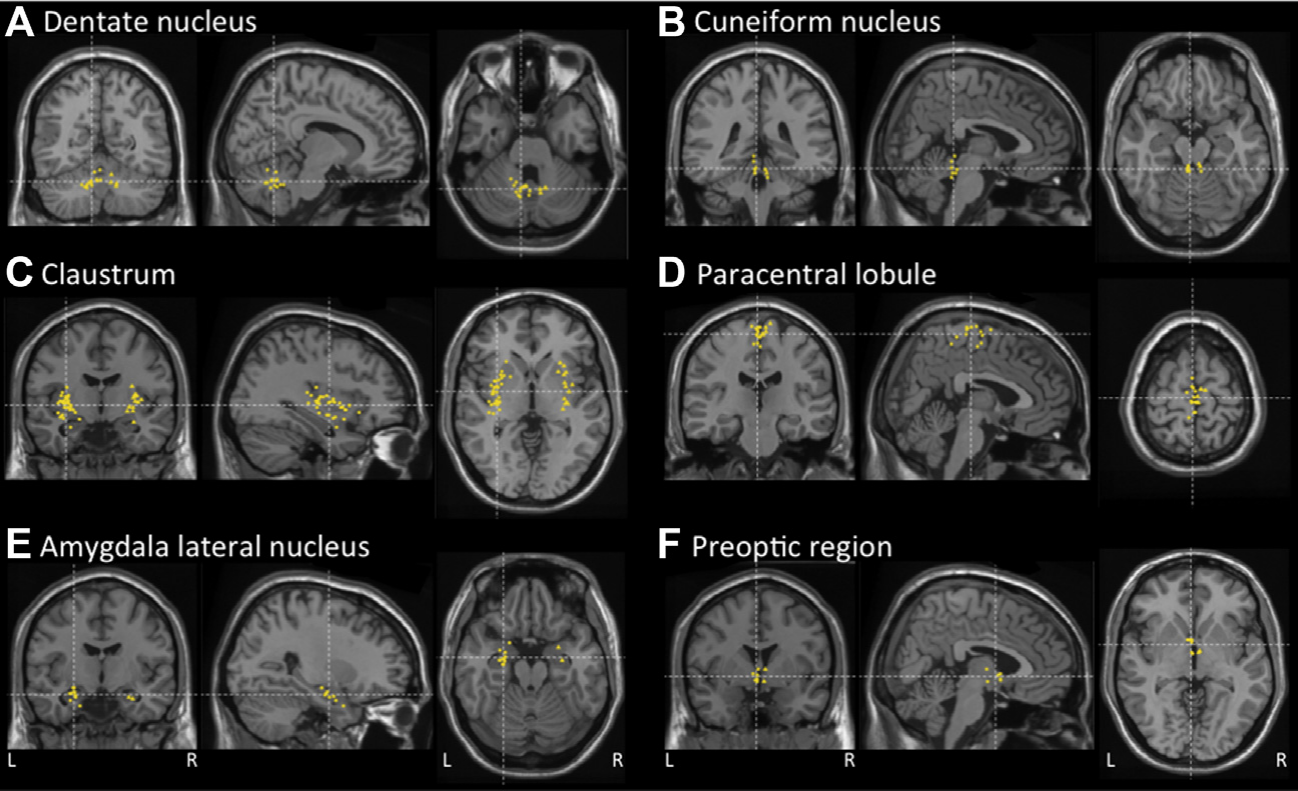
Brain regions with local differential gene expression enrichment for the disruptive behavior and risk-taking behavior factors in coronal, sagittal, and axial views. The locations of the samples of brain tissues that were used to measure gene expression by Hawrylycz *et al.* (31) are projected to a standard Montreal Neurological Institute template brain (Colin27). For every annotation, the figure is centered on the averaged Montreal Neurological Institute coordinates of the brain samples. **(A)** Cerebellum: dentate nucleus. **(B)** Brainstem: cuneiform nucleus. **(C)** Cortex: claustrum. **(D)** Cortex: paracentral lobule. **(E)** Subcortex: lateral amygdaloid nucleus. **(F)** Subcortex: preoptic region.

To pinpoint relevant brain areas associated with DB and RTB more accurately, we proceeded with a local differential gene expression approach using stratified LDSC (31). For DB, we identified significant enrichment after Bonferroni correction exclusively in the dentate nucleus (cerebellum, *Z* = 3.35, *p* = .0004) (Figure 4A; Table S23 in Supplement 2). For RTB, there was also enrichment in the dentate nucleus (cerebellum, *Z* = 3.22, *p* = .0006) and additional enrichment in the cuneiform nucleus (brain stem, *Z* = 4.74, *p* = 1.06 × 10^−6^) (Figure 4B), claustrum (cortex, *Z* = 4.08, *p* = 2.27 × 10^−5^) (Figure 4C), paracentral lobule (cortex, *Z* = 3.35, *p* = .0004) (Figure 4D), lateral amygdaloid nucleus (subcortex, *Z* = 3.12, *p* = .0009) (Figure 4E), and preoptic region (subcortex, *Z* = 3.34, *p* = .0004) (Figure 4F; Table S24 in Supplement 2).

#### Single-Cell Analysis

Finally, we investigated the involvement of specific brain cell types using LDSC. For DB, this revealed enrichment in excitatory neurons of the prefrontal cortex (*Z* = 2.88, *p* = .002) and the hippocampal CA3 region (*Z* = 2.85, *p* = .002) (Table S25 in Supplement 2). For RTB, we found enrichment in excitatory prefrontal cortex neurons (*Z* = 3.14, *p* = .0008) (Table S26 in Supplement 2).

## Discussion

In a bottom-up approach to evaluate overlap in genetic and neurobiological backgrounds underlying externalizing symptoms, we collected 13 publicly available GWAS summary statistics for a range of externalizing phenotypes. Assessment of genetic resemblance indicated a categorization into two externalizing-related factors, one characterized by DB and another by RTB, that together explained 53% of the total genetic variance. Meta-analyzing the factor-specific phenotypes yielded 50 loci for DB and 257 loci for RTB.

Clustering of aggression, angriness, and irritability items in the DB factor fits very well with the general finding that these are highly comorbid behaviors and also cluster together in diagnoses of conduct and oppositional defiant disorder (35). Addition of ADHD aligns with the fact that DBs coexist most commonly with ADHD (36) and with evidence of strong genetic resemblance between ADHD and conduct and oppositional defiant disorder (37). The phenotypes of the RTB factor related to pursuing risk (21). Three RTB traits (general risk tolerance, number of sexual partners, lifetime cannabis use) similarly clustered together in another recent GWAMA (38). Addition of antisocial behavior aligns with the fact that antisocial behavior frequently includes thoughtless, self-centered, and immediately rewarding acts, which are also typical for risk taking. The present RTB phenotypes related to substance use, speeding, and sexual promiscuity are closely linked to social misconduct (38,39). In genomic structural equation modeling, antisocial behavior also loaded on DB, which is concordant with observations from the Hierarchical Taxonomy of Psychopathology consortium that antisocial behavior is a broad construct and also alludes to disruptive phenotypes (1,40).

Assessment of genetic relationships with other traits indicated associations for DB with higher neuroticism and lower conscientiousness and agreeableness, corroborating previous findings (41). For RTB, we found associations with higher extraversion, higher openness to experience, and lower conscientiousness, which is also in line with earlier reports (42). Another notable result was that DB, much more than RTB, showed negative genetic associations with indices of mental and physical health. Previous studies indicated associations with mental and physical health problems for both disruptive-ADHD and risk-taking behaviors (43, 44, 45, 46). This finding of stronger genetic correlations for DB points to the possible distinction that DB phenotypes, characterized by affective instability/irritability and impaired cognitive control, are generally central to mental and physical health problems (44,47), whereas for RTB, mental health problems are more often at the core (45), resulting in risk behaviors, such as substance use, that ultimately affect physical health (46).

In addition, we applied MR to explore causal relationships between the DB factor and smoking and alcohol use. In full agreement with previous findings (15,48), we obtained evidence that DB traits promote substance use (strong evidence for an increased odds of smoking initiation and decreased odds to successfully quit smoking and weak evidence for increased odds of alcohol use disorder). This causal direction is also consistent with expectations for behavioral development, in that DB traits are more likely to occur at younger ages than risk behaviors. Smoking and alcohol use are not yet appropriate and less feasible during early childhood (15,48). However, we also replicated the observation that smoking might causally increase the risk for DB traits. This finding could have important consequences for intervention strategies but needs further inquiry. To distinguish a causal influence from horizontal pleiotropy, i.e., when a genetic variant influences the two traits through independent pathways, dose-response effects should be included in future MR analyses (taking cigarettes per day as the exposure and applying stratification on smoking status).

Biological annotation by gene set analysis and stratified LDSC converged on genetic enrichment in the brain for both DB and RTB traits. An atlas of differential brain region gene expression pointed specifically to the dentate nucleus of the cerebellum, for both DB and RTB, and the cuneiform nucleus of the brainstem, preoptic region of the hypothalamus, lateral nucleus of the amygdala, and the claustrum and paracentral cortical lobule, which were significant for RTB.

Involvement of these brain regions confirms current hypotheses on the neurobiological background of externalizing symptoms but also points to the importance of relatively underrepresented regions. Especially for the medial part of the preoptic hypothalamic region, animal studies indicate an important role in stimulating aggression to facilitate reproduction (49), as well as in modulating mesolimbic activity involved in reward processing (50). The contribution of the amygdala to emotional processing is also generally recognized (51), and there is ample evidence that amygdala–frontal cortex network abnormalities predispose to externalizing behaviors (14), including aggression and risk taking (52,53). In addition, amygdala volume reductions are commonly found in ADHD (54, 55, 56). Furthermore, a recent study showed that GABAergic neurons in the medial amygdala project to the medial preoptic area to regulate reward from social stimuli by controlling the release of dopamine in the nucleus accumbens (57).

The other brain regions have been implicated less frequently, despite previous evidence for associations. For the cerebellar dentate, a relevant role in the development of reinforcement learning relevant to addiction has been indicated (58), and there is evidence that abnormal development of corticocerebellar connections contributes to ADHD (59). The cuneiform nucleus is part of the mesencephalic locomotor control network (60) and related to autonomic fear and stress responses (61,62). The claustrum is richly interconnected with almost all regions of the cerebral cortex and hypothesized to play a role in regulating attention and resilience to distraction (63,64), which links this region to ADHD (65). The anterior section of the paracentral lobule includes motor control regions but has also been linked to executive control function and attention orienting (22,23). Single-cell analysis further elucidated the important role of the CNS for both DB and RTB and pointed to glutamatergic prefrontal neurons for DB and RTB and pyramidal hippocampal neurons for DB. Glutamatergic neurotransmission has been associated with alcohol and tobacco use (33) and RTB (21) before, but more research is needed to address the role of these specific cell types and their functions on the externalizing phenotypes.

Finally, testing for specific biological mechanisms using gene set analysis indicated associations for gene sets primarily related to neuronal development for both DB and RTB. In addition, synaptic functions and transcription regulation were identified for RTB. These three biological mechanisms have been related to a wide range of psychiatric disorders (66) and may play a broad role in behaviors and brain-related traits.

This study comes with a number of limitations. First, the two identified factors DB and RTB combine several distinct phenotypic measures, which inevitably leads to etiologic heterogeneity (reflected by the imperfect genetic correlations). In addition, phenotyping could be as poor as a single question (e.g., about regular use of a substance). Poor and heterogenous phenotyping, with the trade-off of having large sample sizes available, are a common phenomenon in GWAS meta-analyses of single traits (67) or multiple traits (22) and generally results in lower SNP heritability estimates compared with the individual traits, which was also the case here for DB (4%) and RTB (2%). Second, we incorporated three measures on irritability, which seems redundant. However, the mutual genetic correlation between these phenotypes was not perfect (*r*_*g*_ < 1), and therefore, we note that including GWAS statistics of all three phenotypes does provide additional genetic information in GWAMAs. Third, in the MR analyses, the tested effects from smoking and alcohol use to DB did not fully comply with the expected temporal order. The aggression and ADHD variables of DB included children (which amounts to ∼5% of the total number of DB participants). This means that smoking and alcohol use were, for those variables, not valid exposures. In addition, we limited assessment of causal directions to the relationship between the DB factor and smoking and alcohol use. We also demonstrated interesting relationships of DB and RTB with several other traits, such as personality and health characteristics. Given the complexity of the included study cohorts forming the different summary statistics, it was beyond the scope of this study to also test for causal relationships with these traits. Fourth, except for ADHD, the GWAS data in our study related to behavioral extremes that did not exceed the diagnostic threshold. Given previous evidence of genetic heterogeneity between nondiagnosed and diagnosed individuals (68,69), we emphasize that the findings in this study apply in particular to general rather than excessive diagnosed externalizing behaviors. Finally, regarding the biological annotation analyses, we must consider limitations in drawing conclusions about different findings for DB and RTB. DB and RTB are moderately correlated (*r*_*g*_ = 0.33) and therefore not completely independent. In addition, the effective sample size of RTB is ∼3 times larger than that of DB (1,506,537 vs. 523,150), so there is likely more power to detect significant associations for RTB. For example, differential gene expression indicated more brain regions with statistically significant enrichment for RTB, but we note that the same regions also showed relatively increased enrichment among the 211 brain regions tested for DB.

In summary, we extend previous findings involving externalizing behavior and provide further evidence for common genetic architectures, particularly for different DBs and RTBs. Follow-up evaluations of the data in this study indicated genetic relationships with personality traits and mental and physical health behaviors in agreement with the DB versus RTB categorization and highlighted possible bidirectional causal relationships between DB and substance use traits. Biological annotation revealed generally similar gene-brain mappings that mediate the predisposition to comorbid externalizing phenotypes. Possible subtle differences in neurobiological backgrounds between DB and RTB traits may be resolved in future studies based on higher-resolution genetic association and gene expression data that are becoming increasingly available.

## Supplementary Material

Supplement 2

Key Resources Table
