# Supplementary material for: The Genetic and Neural Substrates of Externalizing Behavior
Source: Biol Psychiatry Glob Open Sci. 2021 Oct 6;2(4):389–99. doi: 10.1016/j.bpsgos.2021.09.007 (PMC9616240; doi:10.1016/j.bpsgos.2021.09.007)
Supplement: Supplement 1 [file mmc1.pdf]

The Genetic and Neural Substrates of Externalizing Behavior

Supplement 1

Phenotype selection

To select datasets, we applied the disinhibited externalizing spectrum described by Krueger et al. (1), which connects the psychopathologies of antisocial personality disorder (ASPD), conduct disorder (CD), oppositional deviant disorder (ODD), attention deficit hyperactivity disorder (ADHD), intermittent explosive disorder (IED), and substance use disorders (SUD). Based on this, we collected GWAS summary statistics for 13 externalizing phenotypes (Table 1) originating from four separate studies on antisocial behavior (2), aggression (3), ADHD (4), and lifetime cannabis use (5). In addition, we added five GWAS datasets from a study on the genetic background of risk taking (6). These datasets concerned the phenotypes general risk tolerance, drinks per week, ever smoking, number of sexual partners, and automobile speeding propensity. Furthermore, we identified four items related to angriness and irritability in UK Biobank (7) (UKB Data fields: angry outbursts: 20494; irritability: 1940; being irritable for more than 2 days: 4653; having experienced extreme irritability: 20502). We performed GWAS on each of the UK Biobank items separately using a linear model as implemented in PLINK 2.0 to generate summary statistics (8). Only unrelated participants of UK-ancestry were included in the GWAS, and age, sex and the first 40 genetic principal components were used as covariates. Additionally, a dummy variable was generated to indicate participants genotyped using the UKB BiLEVE array, rather than the Axiom Array. This variable was used as an additional covariate to prevent bias from genotyping platform.

Identification of genetic factor structures

Genetic correlation using LD Score regression

We applied linkage disequilibrium score regression (LDSC) (9,10), to compute genetic correlations between GWASs without bias from possible sample overlap (9). This method regresses the effect sizes of SNPs taken from GWAS summary statistics on the linkage disequilibrium (LD) score and estimates the genetic covariance between the traits from the slope of this regression. Precalculated LD scores from the 1000 Genomes European reference population were obtained from the LD score regression website. Subsequently, the genetic correlation was calculated using HapMap3 SNPs only to ensure high-quality LD score

estimation. Significance of genetic correlation was tested using a Bonferroni corrected p-value threshold (0.05/13 = .0038).

Genomic SEM

The Genomic SEM R-package (11) was used for factor analysis on the GWAS summary statistics. First, we performed exploratory factor analyses (EFA) to examine the number of latent factors and identify which phenotypes load on these factors. Next, we performed confirmatory factor analyses (CFA) to assess which factor structure had the best model fit. To examine the genomic SEM fits, we used the model χ^2 which is estimated based on the sampling covariance matrix of the model residuals, and indicates differences between the model-implied genetic covariance matrix and the empirical genetic covariance matrix (12). In addition, we included the Comparative Fit Index (CFI) and Standardized Root Mean Square Residual (SRMR). CFI compares the fitted model to a model in which all phenotypes are heritable but assumed to be genetically uncorrelated. SRMR examines the standardized root mean squared difference between the model-implied and observed correlations. CFI values ≥ 0.90 and SRMR values < 0.10 are considered an acceptable fit (12). Besides the absolute fit indices, we examined relative fit indices to select a parsimonious model that approximates the empirical genetic covariance matrix not worse than a complex model. The Akaike Information Criterion (AIC) was used to compare models. To attain semi-independent datasets for EFA and CFA, we split the included datasets into a set containing even chromosomes, for EFA, and a set containing odd chromosomes, for CFA (13).

Multivariate N-Weighted Genome-Wide Association Meta-Analysis (*N-GWAMA*)

N-weighted GWAMA is based on quantifying the dependence between summary statistics using the pair-wise cross-trait LD-score intercepts (CTI). The CTI is similar to the covariance between the test statistics of the traits between two univariate GWASs. Using the estimated covariances between effect sizes, the univariate GWASs are meta-analysed by calculating the multivariate test statistic per SNP. From the GWAMA results, independent significant SNPs were defined as being in low LD ($r^2 < 0.1$), and a minimum distance between two independent loci was set at 250 kb using FUMA (14).

Genetic relationships with other traits

We computed pairwise genetic correlations using LDSC (9,10) for our identified factors with 61 additional phenotypes subdivided in the categories: mental health, cognition and

socioeconomic status (SES), personality, social, substance use, cardiovascular disease risk, physical health, anthropomorphic, and reproduction. The genome-wide LD information was based on European populations from the HapMap 3 (1,290,028 SNPs) reference panel, and all GWAS summary statistics were filtered to these SNPs. The level of significance for the genetic correlations was set at $0.05/61 = 8.20 \times 10^{-04}$.

Mendelian Randomization

To test for causal relations between a cluster of disruptive behaviors identified in our study and substance use behaviors. using Mendelian Randomization, we obtained additional and independent summary level data from the most recent and largest GWASs for smoking (initiation, n=848,460, cigarettes per day, n=216,590, and smoking cessation, n=378,249) (15) and alcohol (drinks per week, n=630,154 (15), alcohol use disorder, n= 46,568 (16)). We prevented sample overlap with the datasets in our meta-analyses by excluding UK Biobank from the obtained smoking & alcohol per week GWASs. There remained a small amount of sample overlap between the aggression GWAS (3) included in our disruptive behavior cluster and the smoking/alcohol GWASs, but this is unlikely to cause bias given that aggression is only one of six phenotypes included in the cluster. Note that we were only able to test causal effects with smoking initiation as exposure, and not cigarettes per day or smoking cessation, because the latter two GWASs were performed in (former) smokers only meaning any Mendelian Randomization analysis using these genetic associations would need to be stratified on smoking status.

Mendelian Randomization (MR) is a method to assess if individual differences in a certain human trait (an exposure such as disruptive behavior in our study) causally influence differences in another trait (an outcome such as smoking) (17). MR uses information on genetic variants influencing human traits that can nowadays be obtained through genome-wide association studies (GWAS). It involves testing whether genetic variants associated with an exposure (e.g. DB) are also able to predict differences in an outcome trait (e.g., smoking). The genetic variants are used as measures of individual differences in exposure, i.e. as a proxy. Given random segregation of genetic variants (Mendel's 2nd law), the genetic variants should not be associated with common confounders such as socio-economic status. This allows for much less biased causal inference (18). Three assumptions underly MR, which propose that the genetic instrument is, 1: robustly predictive of the exposure, 2: not associated with confounders, and 3: not associated directly with the outcome, other than through its potential

causal effect on the exposure. Horizontal pleiotropy, where (some of) the genetic variants in the instrument directly affect multiple traits, could lead to assumptions 2 and 3 being violated. To maximise reliability, we combined a diverse set of complementary MR methods. If an effect is consistent across these methods, this indicates robust evidence for a causal effect.

The main MR analysis is Inverse Variance Weighted regression (IVW). IVW computes the ratio of the SNP-exposure association (from the exposure GWAS) and the SNP-outcome association (from the outcome GWAS) and combines this for all SNPs (19). We applied six sensitivity methods. First, weighted median regression, which provides a reliable estimate when ≥50% of the weight of the instrument comes from valid (i.e., unbiased) SNPs (20). Second, weighted mode regression, which gives a reliable estimate when the causal effect that is most common is consistent with the true causal effect (21). Third, MR-Egger, which relaxes the main assumptions of MR, but only provides a reliable causal estimate when the INSIDE assumption (instrument strength independent of direct effects) holds (20). Conveniently, MR-Egger allows a test for horizontal pleiotropy in the form of its intercept. Fourth, GSMR (Generalised Summary-data-based Mendelian Randomisation), which takes into account very low levels of LD between SNPs included in the instrument, thereby attaining relatively high statistical power. GSMR includes a filtering step which removes outliers based on their effect size (HEIDI-filtering) (22). Fifth, Steiger filtering, which calculates the amount of variance that each SNP explains in both the exposure and the outcome and then tests if the explained variance is higher for the exposure than the outcome. If not – i.e., a SNP explains more variance in the outcome – it implies reverse causation (23). After excluding SNPs that don't survive Steiger filtering, the main MR analyses were repeated. Sixth and final, MR-PRESSO (Pleiotropy Residual Sum and Outlier) was applied to identify any outliers and, if detected, to test whether outlier-corrected IVW analyses provided consistent results (24).

Tissue type associations

Using MAGMA version 1.08 (25), we investigated tissue-specific gene-expression values as gene properties (i.e., continuous values for all genes) using 53 tissues from the Genotype-Tissue Expression (GTEx) project v7 (http://www.gtexportal.org/home/datasets)(26). The expression values of all genes were Winsorized at 50 reads per kilobase of transcript per million reads mapped (RPKM) and subsequently log2 transformed with pseudocount 1. The tissue-type associations were corrected for dependencies between genes and for confounding effects of gene size, gene density, mean minor allele count in the gene and per-gene sample size by

adding these variables and their log values as covariates to the regression model. A Bonferroni correction was applied to correct for multiple testing.

Gene-set associations

In competitive gene-set analyses using MAGMA 1.08, we tested whether the genes in a gene set are more strongly associated with the factor phenotypes than the other genes in the genome. Thereby we corrected for the baseline level of association present in the dataset which is especially important for polygenic traits because polygenicity can induce an association of any random gene set of sufficient size. We tested all Gene Ontology (GO) gene sets (n = 5917) and canonical pathways (n = 1329) from the molecular signature database MsigDB v6.2 (http://software.broadinstitute.org/gsea/msigdb) (27). The GO gene sets contain genes annotated by the same GO term and cover biological processes, cellular components, and molecular functions. The canonical pathways are representations of biological processes, compiled by domain experts.

Conditional analyses for tissue types and gene sets

In conditional analyses using MAGMA 1.08 (25), we evaluated redundancy between associations. This provides additional insight because genes can have multiple biological functions that often correlate across genes, which may cause confounding effects in gene-set analyses. In addition, many gene sets overlap due to a hierarchical structure of the database.

A stepwise procedure was followed (28). In the first step, we investigated the overlap in expression levels of the identified tissue types of GTEx in a forward selection procedure. We started by selecting the tissue type with the strongest association with the phenotype, on which the second most strongly associated tissue type was conditioned (in addition to average expression levels that were already included as a covariate in the initial tissue type analysis). In every next step, we repeated this by conditioning on those tissue types that remained nominal significantly associated ($P<0.05$) in the previous steps. The remaining tissue types will reflect independent association signals with the factors. In the second step of the conditional analyses, we corrected the GO and canonical pathway gene sets for general confounding of average gene expression levels and the tissue types that we identified in the previous step. In the third step, we performed the same forward selection procedure described in step 1 for the gene sets that remained nominally significant after step 2. These associations were conditioned on the tissues included in step 2 as well.

Stratified LD-Score Regression of tissue types

To investigate which tissues and cell types are enriched for the identified factors, we used two approaches. We first grouped 54 tissue types of the GTEx Consortium in 10 broad tissue-type groups: adrenal/pancreas, CNS, cardiovascular, connective/bone, gastrointestinal, immune/hematopoietic, kidney, liver, skeletal muscle and 'other'. Next, we examined enrichment of regions of the genome for 220 cell-type-specific annotations (33 brain and 187 non-brain annotations) for four specific histone marks (H3K4me1, H3K4me3, H3K27ac and H3K9ac) in the 54 tissues (29). We started with a baseline model, consisting of 54 tissue categories and a category containing all SNPs. For every annotation, we then added the cell-type specific annotation separately to the baseline model to control for overlap in the 54 functional categories. For all resulting models (10 in the first round, 220 in the second), we applied stratified LD score regression to test whether the annotation contributes significantly to the per-SNP heritability (29).

Stratified LD score regression of local gene expression across the human brain.

We downloaded the normalized and QC'ed gene expression measured in an anatomically comprehensive set of brain regions from http://www.brain-map.org/. The data contains 3707 measurements across 6 adult donated human brains. The procedures used to measure standardized gene expression across the brains are described in Hawrylycz et al. (30). Based on these data we computed differential gene expression for 48154 probes which map to 20724 unique genes (probes which did not map to genes were omitted). Conform Baselmans et al. (31), we considered differential gene expression across 210 regions subdivided in four gross anatomical regions (brainstem, cortex, subcortical structures and cerebellum). For each gene in each region a t-test was performed, testing the difference in standardized expression between the region in question and all other brain regions present in an anatomical region (e.g. brainstem). The top 10% of probes ranked in terms of t-statistic per region were retained. The unique genes mapped to this set of probes were extracted (mapping ~2900-3500 genes to each region). A partitioned LD score with respect to the genomic regions spanned by these genes (using gencode v19 as a reference), and a 100 kilobase window around each gene, was computed. The heritability of DB and RTB was partitioned across the 54 baseline annotations developed by Finucane et al. (32) and each of the brain regions located in an anatomical separate region (the regions are considered separately).

Stratified LD score regression of brain cell types

To determine the differential expression of genes in brain cell types studied by Habib et al. (33), we first computed the average gene count per specific cell type and calculated the standardized expression in all other cell types. The differential expression was then quantified by dividing the relative expression in one cell type over the expression in all other cell types. Subsequently, we used stratified LD score regression to examine which SNPs are expressed in one of the types of neurons using the same procedure (29,31). For these tests, we applied Bonferroni adjustment for multiple comparisons.

Supplementary References

1. Krueger RF, Kotov R, Watson D, Forbes MK, Eaton NR, Ruggero CJ, *et al.* (2018): Progress in achieving quantitative classification of psychopathology. *World Psychiatry* 17: 282–293.
2. Tielbeek JJ, Johansson A, Polderman TJC, Rautiainen M-R, Jansen P, Taylor M, *et al.* (2017): Genome-Wide Association Studies of a Broad Spectrum of Antisocial Behavior. *JAMA Psychiatry* 74: 1242–1250.
3. Pappa I, St Pourcain B, Benke K, Cavadino A, Hakulinen C, Nivard MG, *et al.* (2016): A Genome-Wide Approach to Children's Aggressive Behavior: The EAGLE consortium. *Am J Med Genet* 171: 562–572.
4. Demontis D, Walters RK, Martin J, Mattheisen M, Als TD, Agerbo E, *et al.* (2019): Discovery of the first genome-wide significant risk loci for attention deficit/hyperactivity disorder. *Nat Genet* 51: 63–75.
5. Pasman JA, H Verweij KJ, Gerring Z, Stringer S, Sanchez-Roige S, Treur JL, *et al.* (2018): GWAS of lifetime cannabis use reveals new risk loci, genetic overlap with psychiatric traits, and a causal influence of schizophrenia. *Nat Neurosci* 21: 1161–1170.
6. Karlsson Linnér R, Biroli P, Kong E, Meddens SFW, Wedow R, Fontana MA, *et al.* (2019): Genome-wide association analyses of risk tolerance and risky behaviors in over 1 million individuals identify hundreds of loci and shared genetic influences. *Nat Genet* 51: 245–257.
7. Sudlow C, Gallacher J, Allen N, Beral V, Burton P, Danesh J, *et al.* (2015): UK Biobank: An Open Access Resource for Identifying the Causes of a Wide Range of Complex Diseases of Middle and Old Age. *PLOS Med* 12: e1001779.
8. Purcell S, Neale B, Todd-Brown K, Thomas L, Ferreira MAR, Bender D, *et al.* (2007): PLINK: A tool set for whole-genome association and population-based linkage analyses. *Am J Hum Genet* 81: 559–575.
9. Bulik-Sullivan BK, Loh P-R, Finucane HK, Ripke S, Yang J, Schizophrenia Working Group of the Psychiatric Genomics Consortium SWG of the PG, *et al.* (2015): LD Score regression distinguishes confounding from polygenicity in genome-wide association studies. *Nat Genet* 47: 291–5.
10. Bulik-Sullivan BK, Finucane HKK, Anttila V, Gusev A, Day FRR, Loh P-R, *et al.* (2015): An atlas of genetic correlations across human diseases and traits. *Nat Genet* 47: 1236–41.
11. Grotzinger AD, Rhemtulla M, De Vlaming R, Ritchie SJ, Mallard TT, David Hill W, *et*

al. (2018): Genomic SEM Provides Insights into the Multivariate Genetic Architecture of Complex Traits. *BioRxiv*. https://doi.org/10.1101/305029

12. Grotzinger AD, Rhemtulla M, de Vlaming R, Ritchie SJ, Mallard TT, Hill WD, *et al.* (2019): Genomic structural equation modelling provides insights into the multivariate genetic architecture of complex traits. *Nat Hum Behav* 3: 513–525.
13. Baselmans BML, van de Weijer MP, Abdellaoui A, Vink JM, Hottenga JJ, Willemsen G, *et al.* (2019): A Genetic Investigation of the Well-Being Spectrum. *Behav Genet* 49: 286–297.
14. Watanabe K, Taskesen E, Van Bochoven A, Posthuma D (2017): Functional mapping and annotation of genetic associations with FUMA. *Nat Commun* 8: 1826.
15. Liu M, Jiang Y, Wedow R, Li Y, Brazel DM, Chen F, *et al.* (2019): Association studies of up to 1.2 million individuals yield new insights into the genetic etiology of tobacco and alcohol use. *Nat Genet* 51: 237–244.
16. Walters RK, Polimanti R, Johnson EC, McClintick JN, Adams MJ, Adkins AE, *et al.* (2018): Transancestral GWAS of alcohol dependence reveals common genetic underpinnings with psychiatric disorders. *Nat Neurosci* 21: 1656–1669.
17. Lawlor DA, Harbord RM, Sterne JAC, Timpson N, Smith GD (2008): Mendelian randomization: Using genes as instruments for making causal inferences in epidemiology. *Stat Med* 27: 1133–1163.
18. Treur JL, Munafò MR, Logtenberg E, Wiers RW, Verweij KJH (2021): Using Mendelian randomization analysis to better understand the relationship between mental health and substance use: a systematic review. *Psychol Med* 1–32.
19. Davies NM, Holmes M V., Davey Smith G (2018): Reading Mendelian randomisation studies: A guide, glossary, and checklist for clinicians. *BMJ* 362: k601.
20. Bowden J, Smith GD, Burgess S (2015): Mendelian randomization with invalid instruments: Effect estimation and bias detection through Egger regression. *Int J Epidemiol* 44: 512–525.
21. Hartwig FP, Smith GD, Bowden J (2017): Robust inference in summary data Mendelian randomization via the zero modal pleiotropy assumption. *Int J Epidemiol* 46: 1985–1998.
22. Zhu Z, Zheng Z, Zhang F, Wu Y, Trzaskowski M, Maier R, *et al.* (2018): Causal associations between risk factors and common diseases inferred from GWAS summary data. *Nat Commun* 9: 224.
23. Hemani G, Tilling K, Davey Smith G (2017): Orienting the causal relationship between

imprecisely measured traits using GWAS summary data. *PLoS Genet* 13: e1007081.

24. Verbanck M, Chen CY, Neale B, Do R (2018): Detection of widespread horizontal pleiotropy in causal relationships inferred from Mendelian randomization between complex traits and diseases. *Nat Genet* 50: 693–698.
25. de Leeuw CA, Mooij JM, Heskes T, Posthuma D (2015): MAGMA: Generalized Gene-Set Analysis of GWAS Data. *PLoS Comput Biol* 11: e1004219.
26. GTEx Consortium (2015): The Genotype-Tissue Expression (GTEx) pilot analysis: multitissue gene regulation in humans. *Science* 348: 648–660.
27. Subramanian A, Tamayo P, Mootha VK, Mukherjee S, Ebert BL, Gillette M a, *et al.* (2005): Gene set enrichment analysis: a knowledge-based approach for interpreting genome-wide expression profiles. *Proc Natl Acad Sci U S A* 102: 15545–15550.
28. de Leeuw CA, Stringer S, Dekkers IA, Heskes T, Posthuma D (2018): Conditional and interaction gene-set analysis reveals novel functional pathways for blood pressure. *Nat Commun* 9: 3768.
29. Finucane HK, Reshef YA, Anttila V, Slowikowski K, Gusev A, Byrnes A, *et al.* (2018): Heritability enrichment of specifically expressed genes identifies disease-relevant tissues and cell types. *Nat Genet* 50: 621–629.
30. Hawrylycz MJ, Lein ES, Guillozet-Bongaarts AL, Shen EH, Ng L, Miller JA, *et al.* (2012): An anatomically comprehensive atlas of the adult human brain transcriptome. *Nature* 489: 391–399.
31. Baselmans BML, Jansen R, Ip HFF, Dongen J Van, Abdellaoui A, Weijer MP Van De, *et al.* (2019): Multivariate genome-wide analyses of the well-being spectrum. *Nat Genet* 51: 445–451.
32. Finucane HK, Bulik-Sullivan B, Gusev A, Trynka G, Reshef Y, Loh P-R, *et al.* (2015): Partitioning heritability by functional annotation using genome-wide association summary statistics. *Nat Genet* 47: 1228–1235.
33. Habib N, Avraham-Davidi I, Basu A, Burks T, Shekhar K, Hofree M, *et al.* (2017): Massively parallel single-nucleus RNA-seq with DroNc-seq. *Nat Methods* 14: 955–958.

Supplementary Figures

Figure S1. Scatterplot for two-sample Mendelian randomization analyses

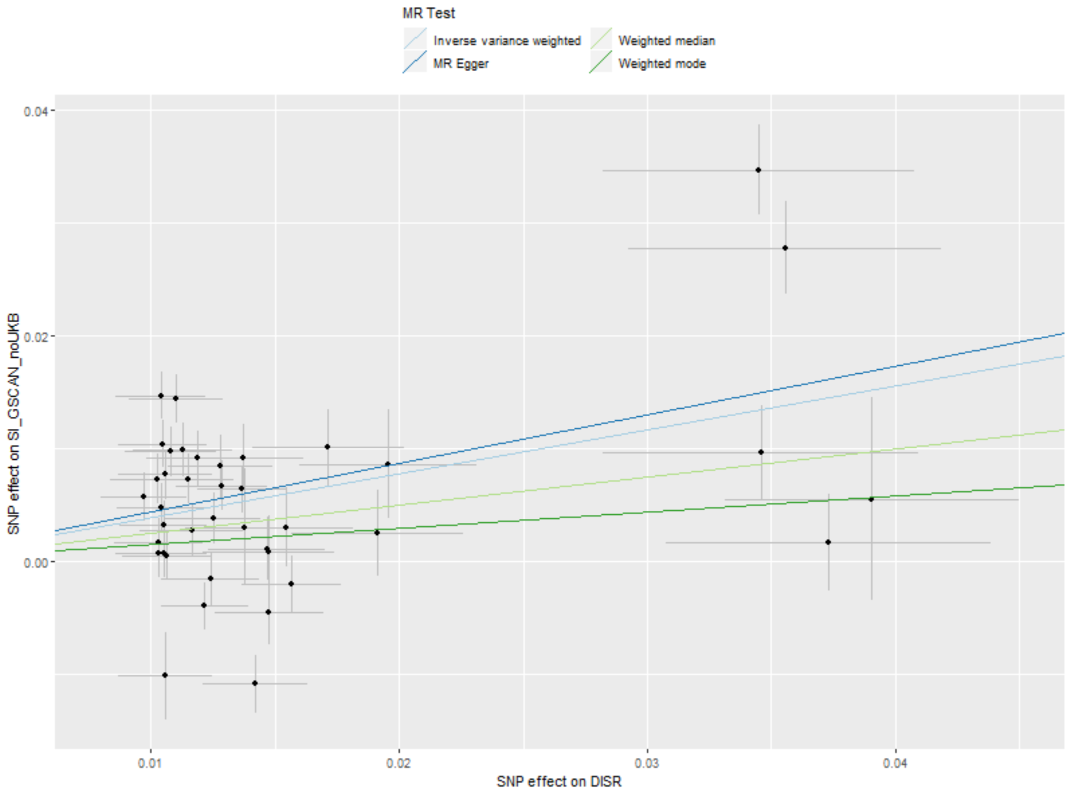

A. From disruptive behavior to smoking initiation.

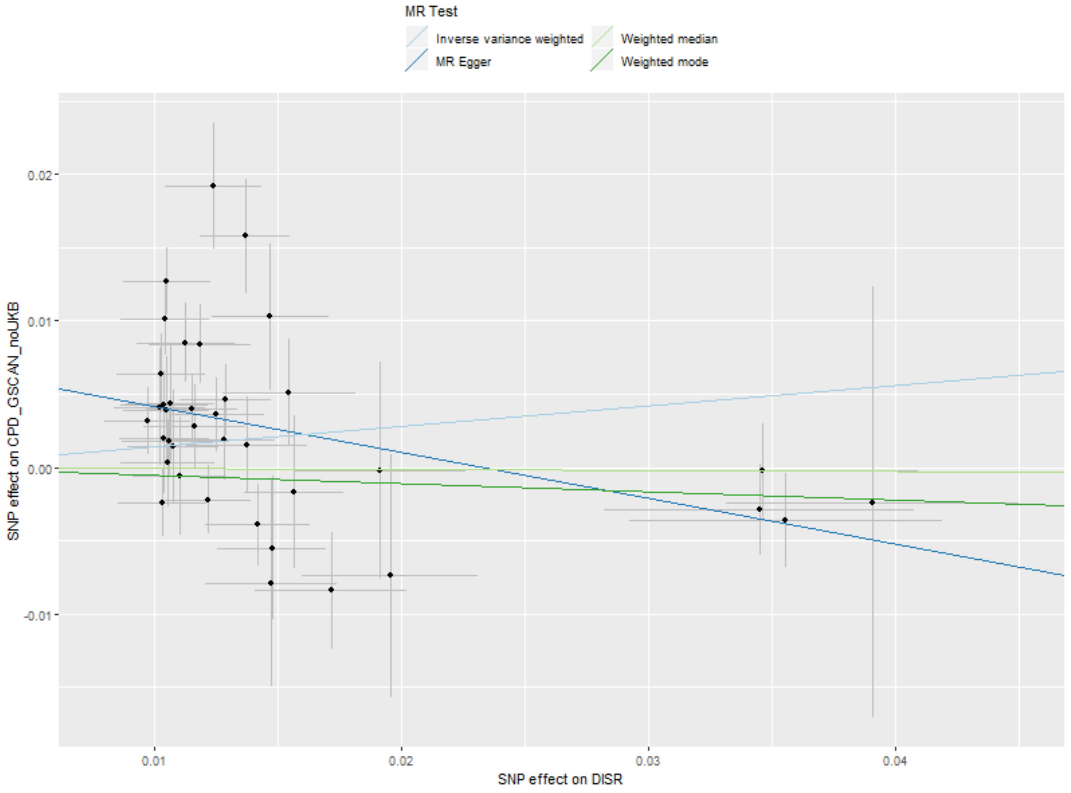

B. From disruptive behavior to cigarettes smoked per day.

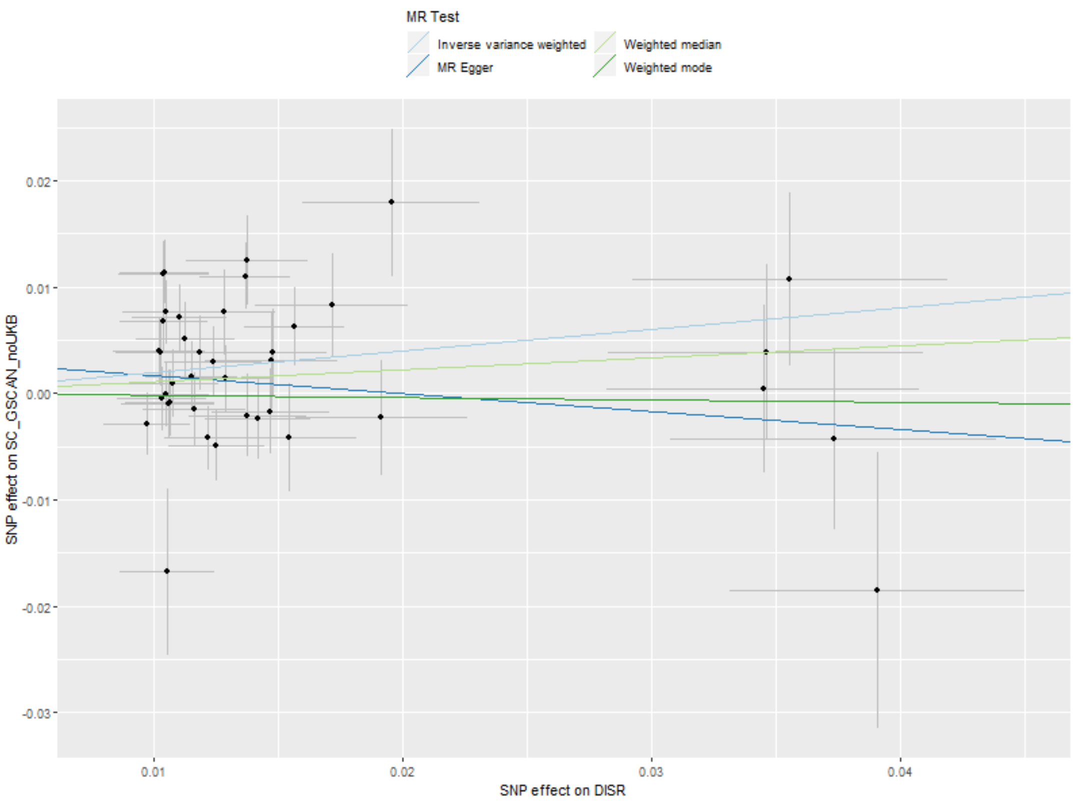

C. From disruptive behavior to smoking cessation.

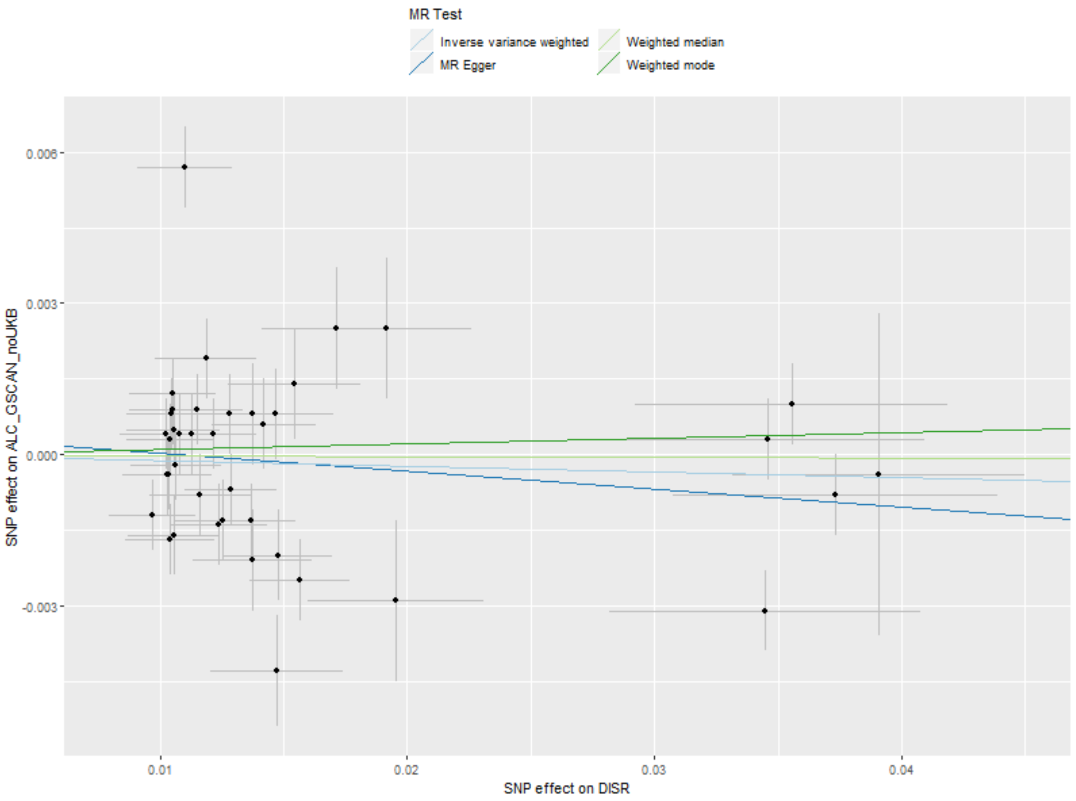

D. From disruptive behavior to alcoholic drinks per week.

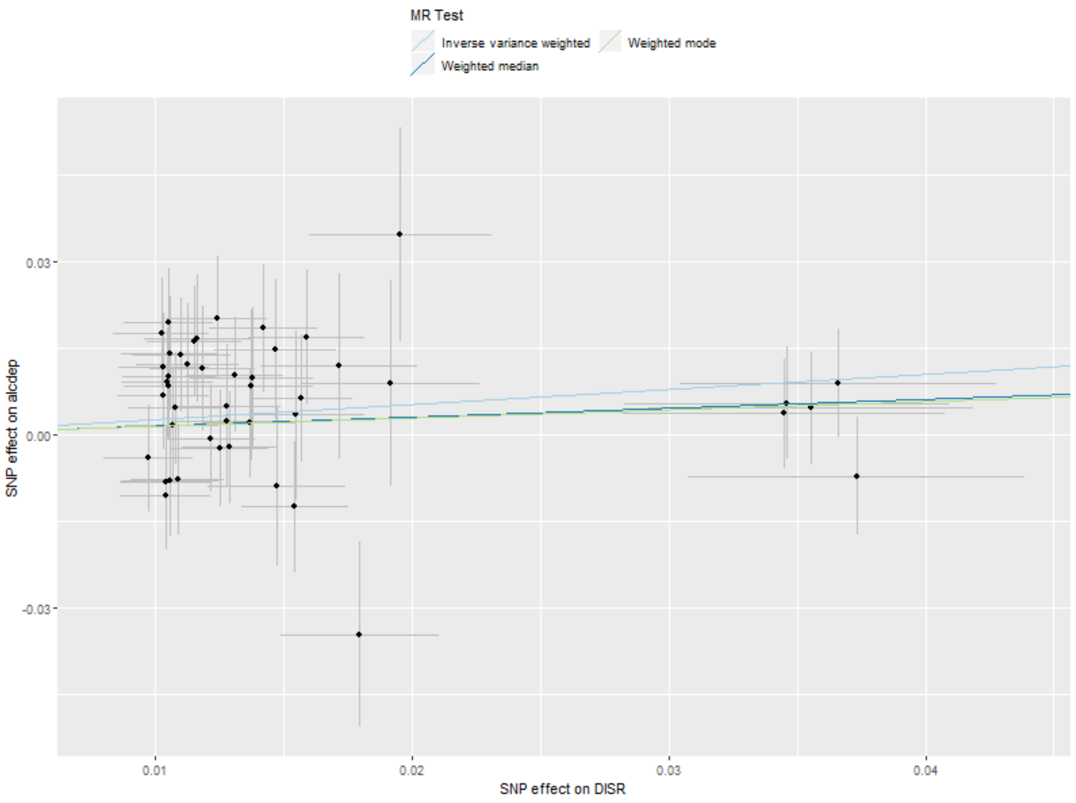

E. From disruptive behavior to alcohol use disorder.

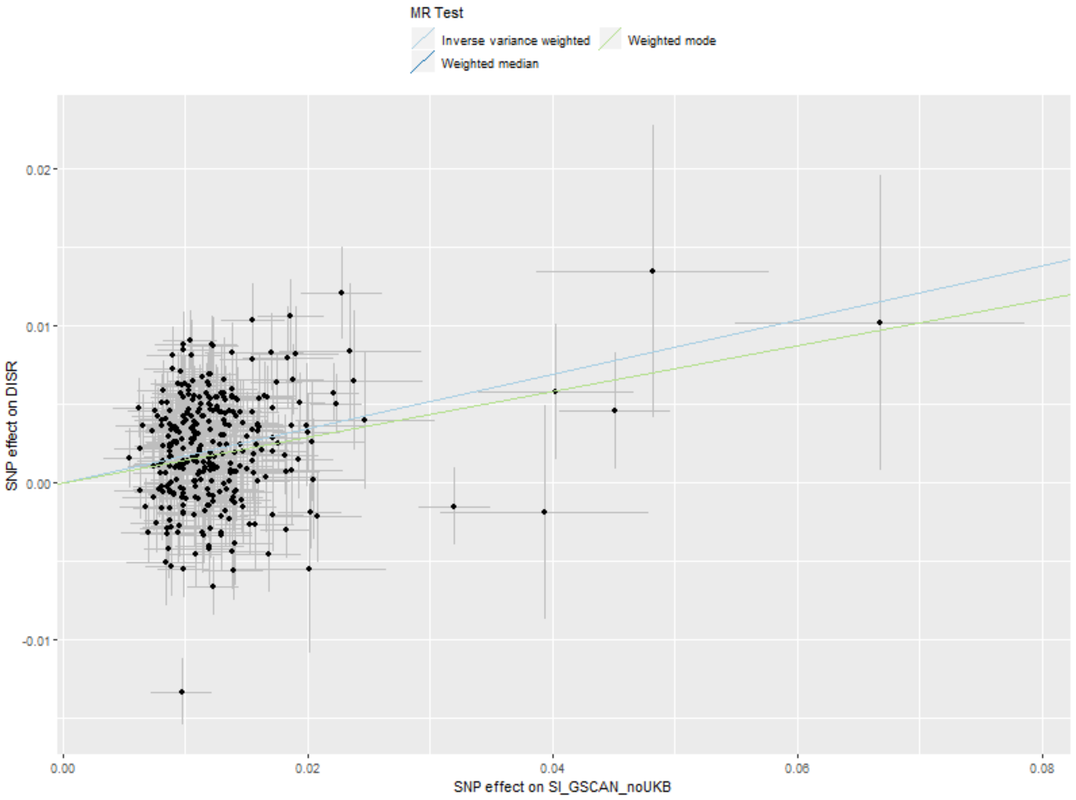

F. From smoking initiation to disruptive behavior.

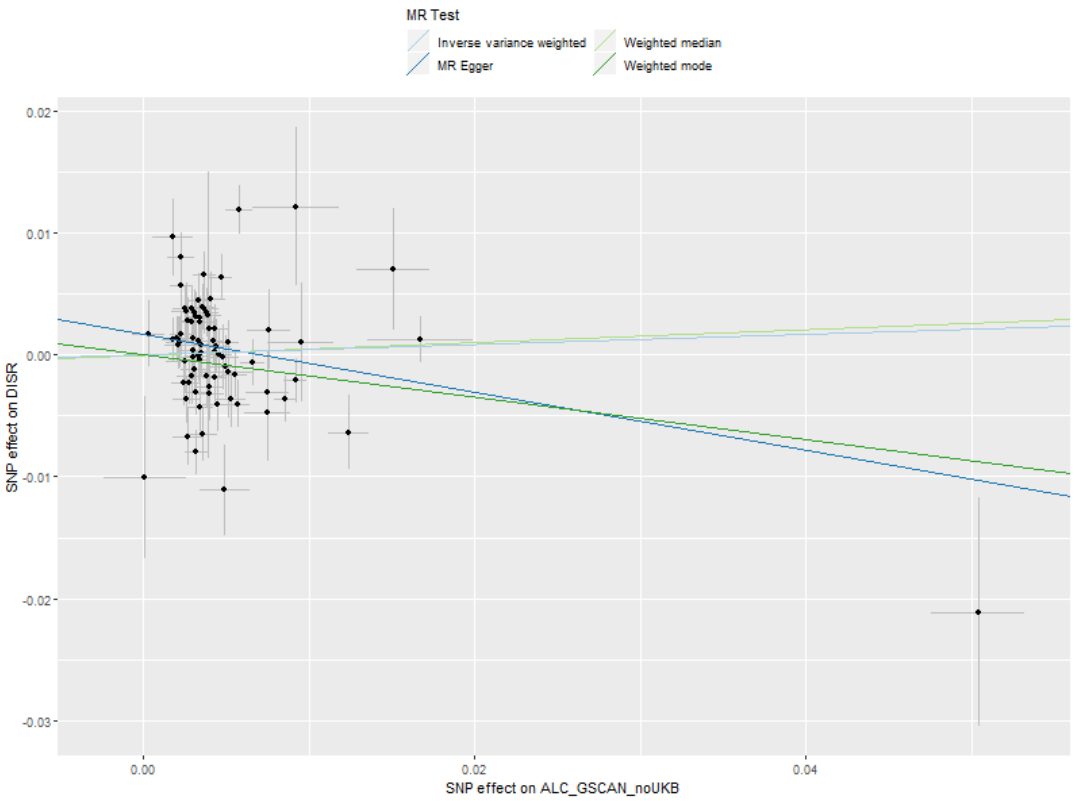

G. From alcoholic drinks per week to disruptive behavior.

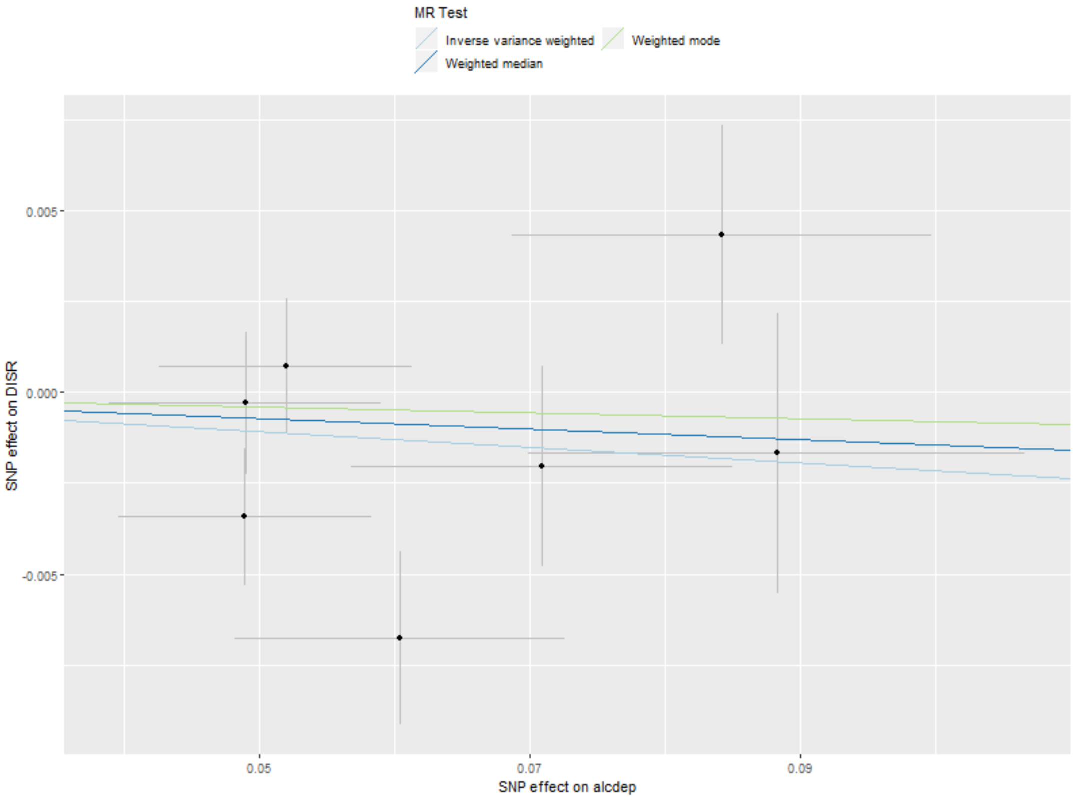

H. From alcohol use disorder, p-value threshold 5E-08, to disruptive behavior.

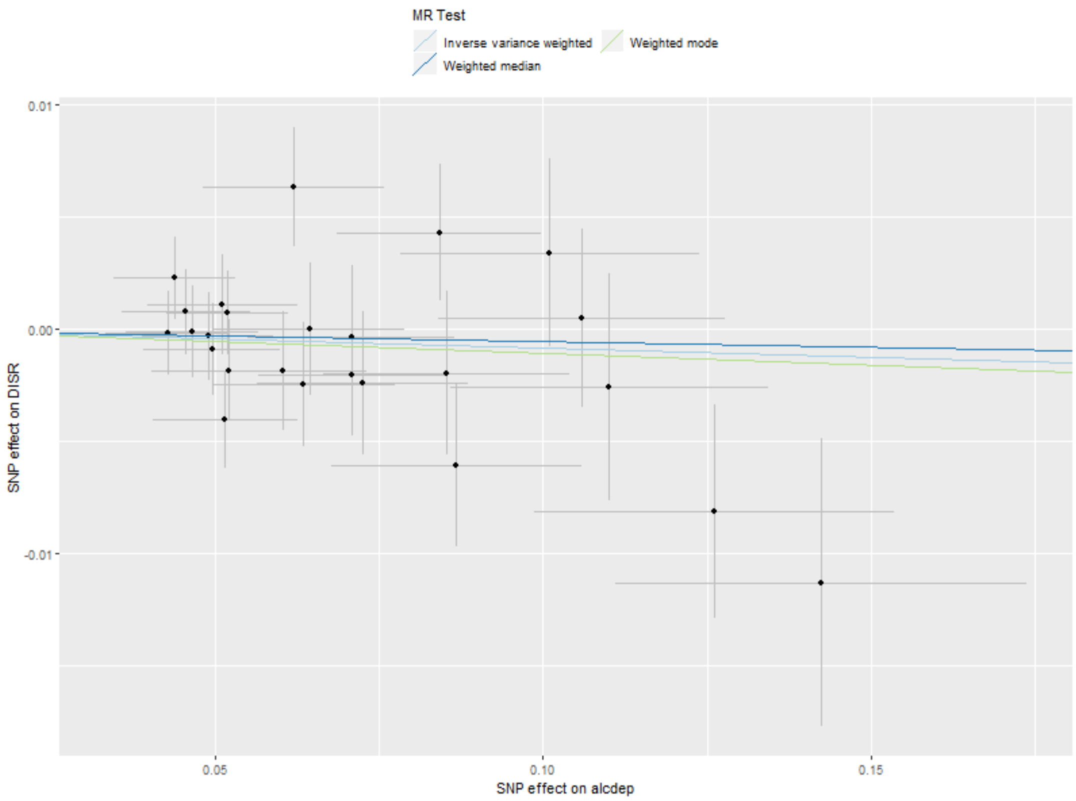

I. From alcohol use disorder, p-value threshold 1E-05, to disruptive behavior.

Figure S2. Leave-one-out analysis for Inverse Variance Weighted (IVW) regression, two-sample Mendelian randomization analyses

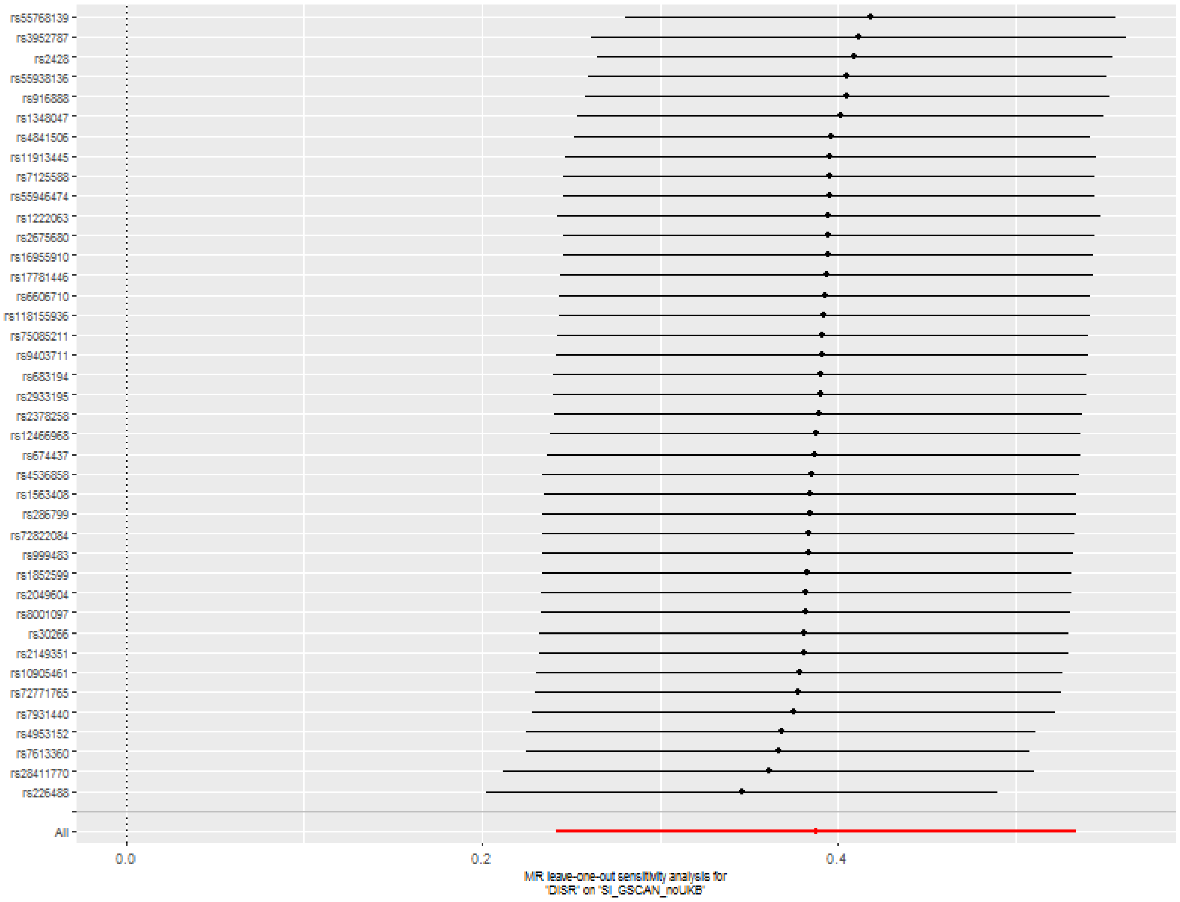

A. From disruptive behavior to smoking initiation.

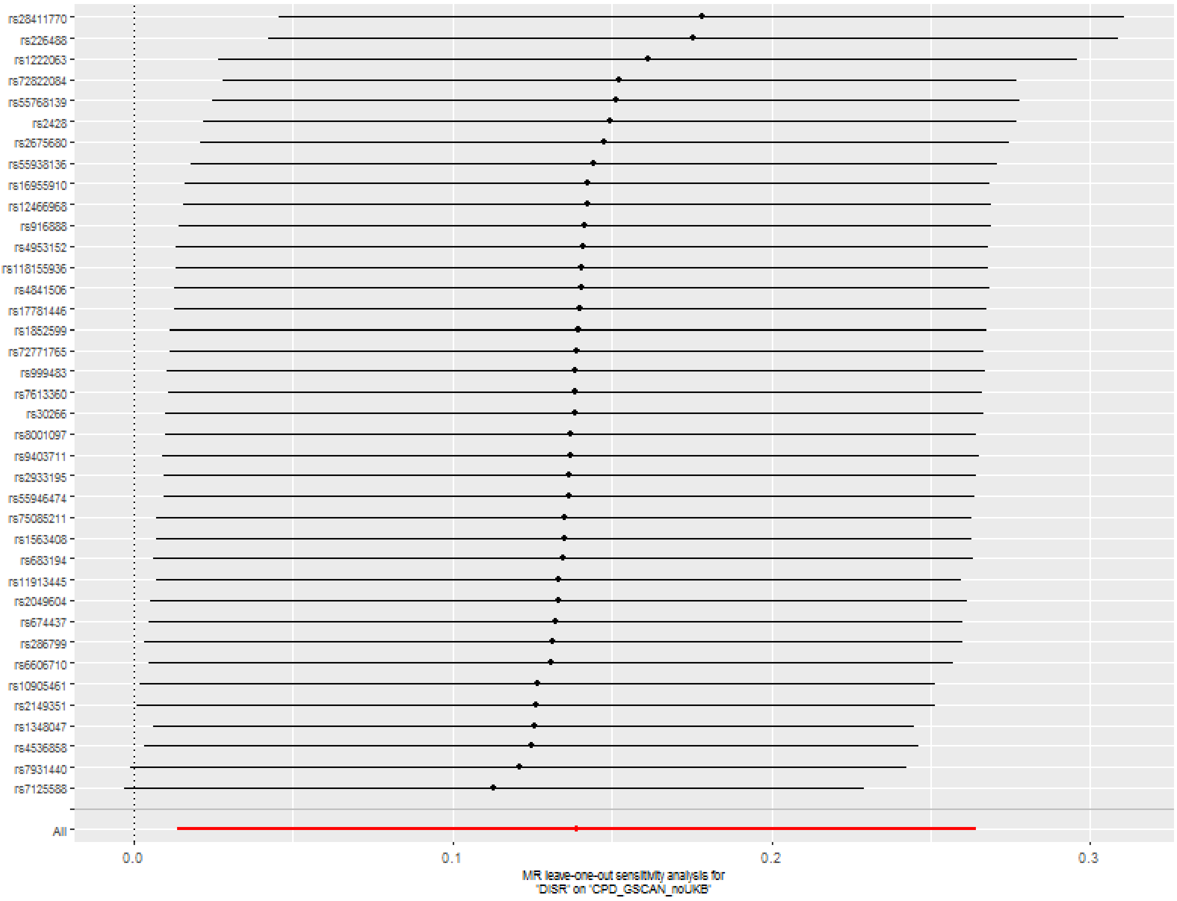

B. From disruptive behavior to cigarettes smoked per day.

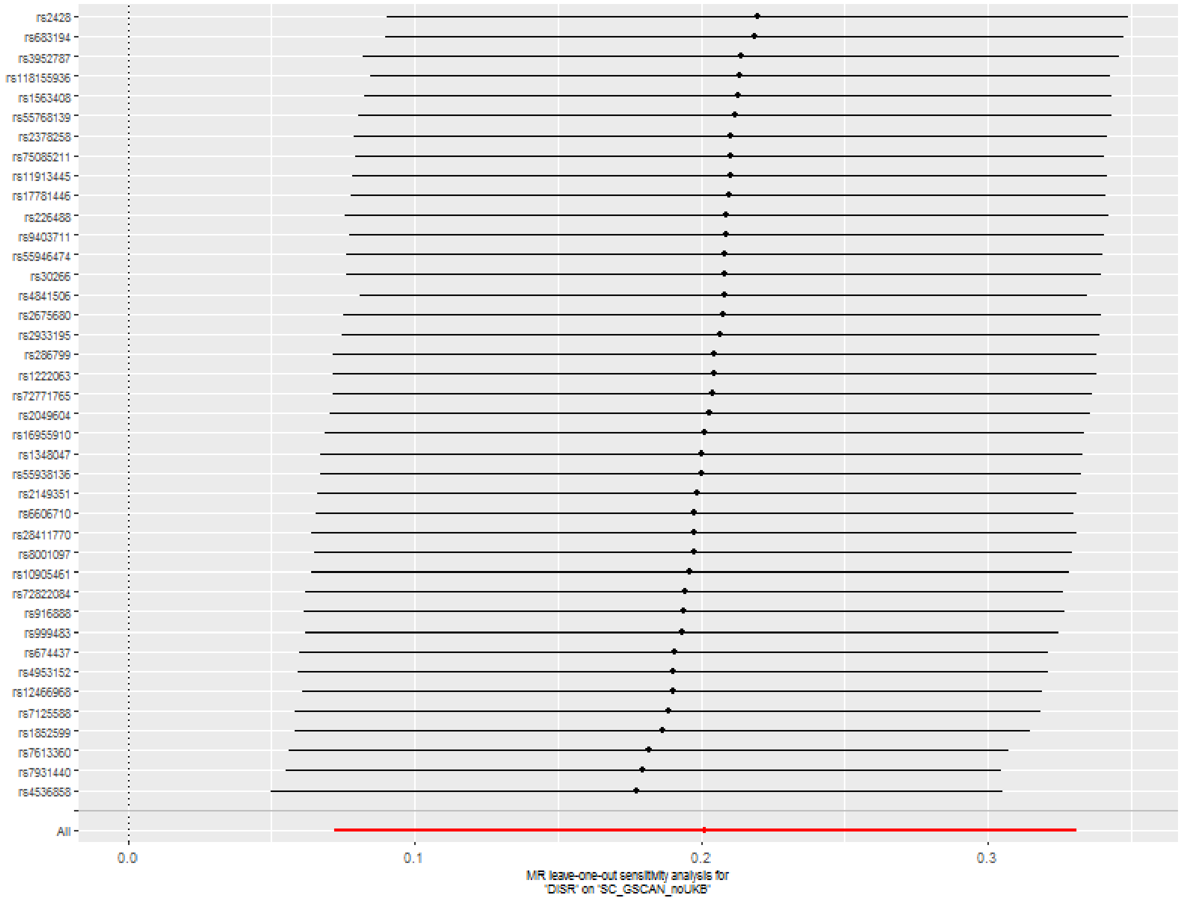

C. From disruptive behavior to smoking cessation.

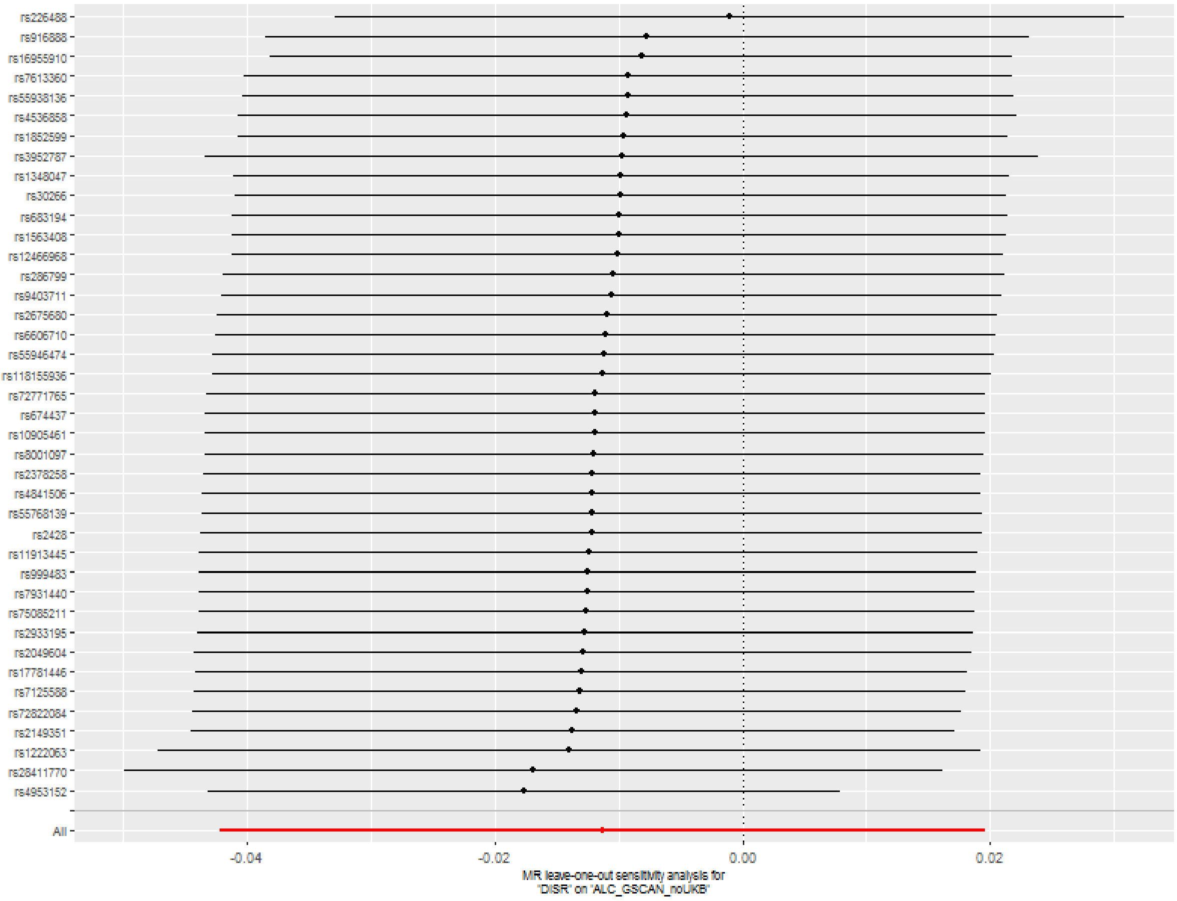

D. From disruptive behavior to alcohol drinks per week.

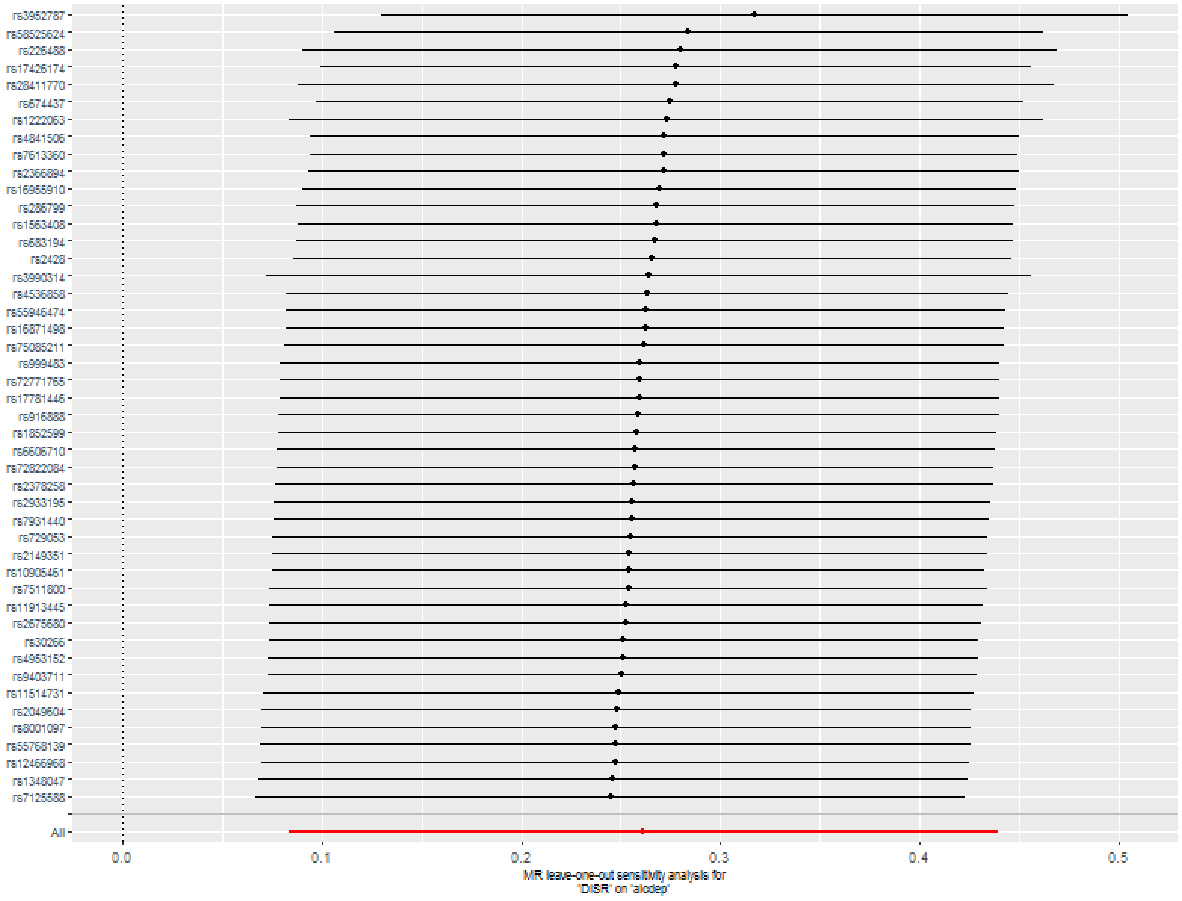

E. From disruptive behavior to alcohol use disorder.

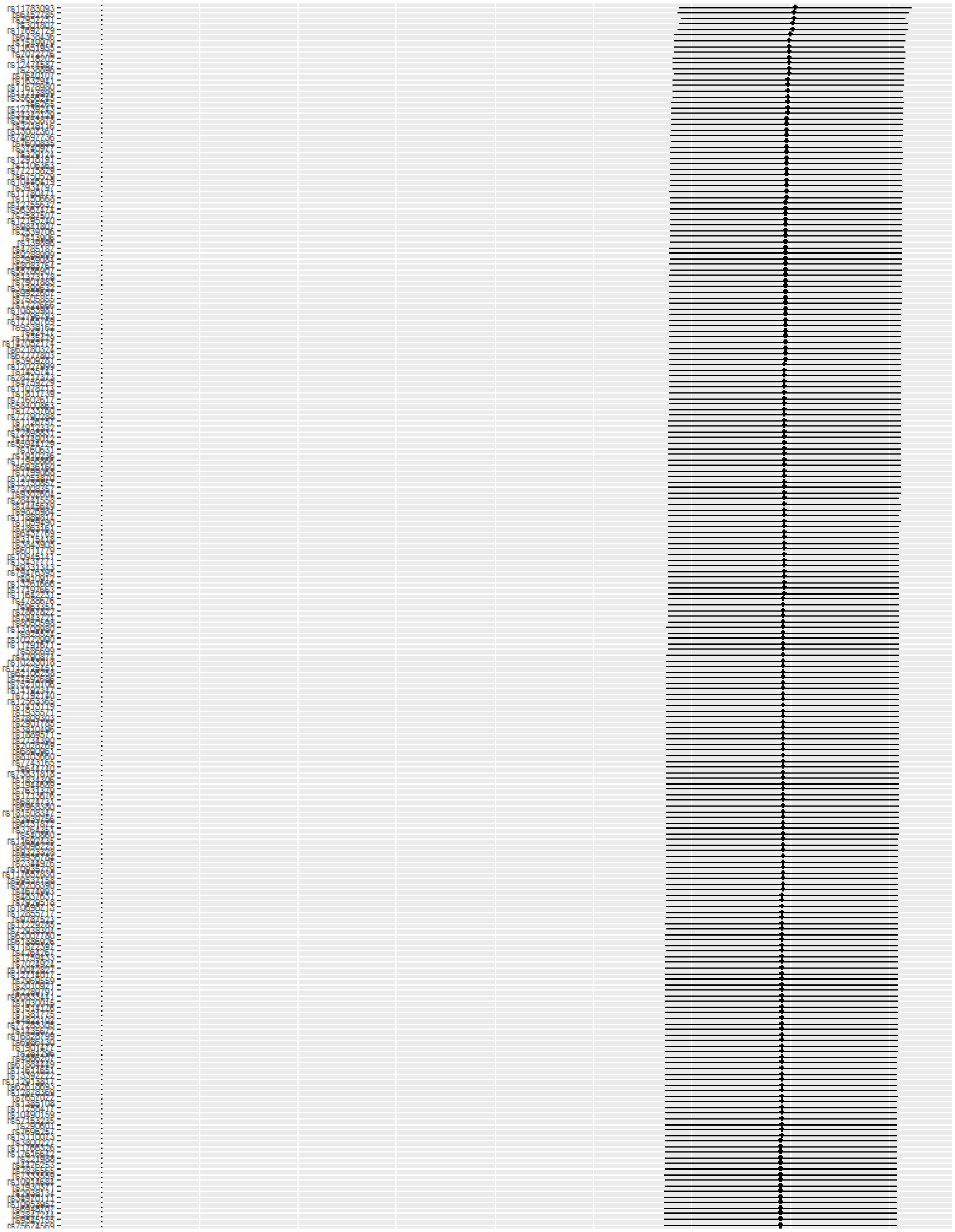

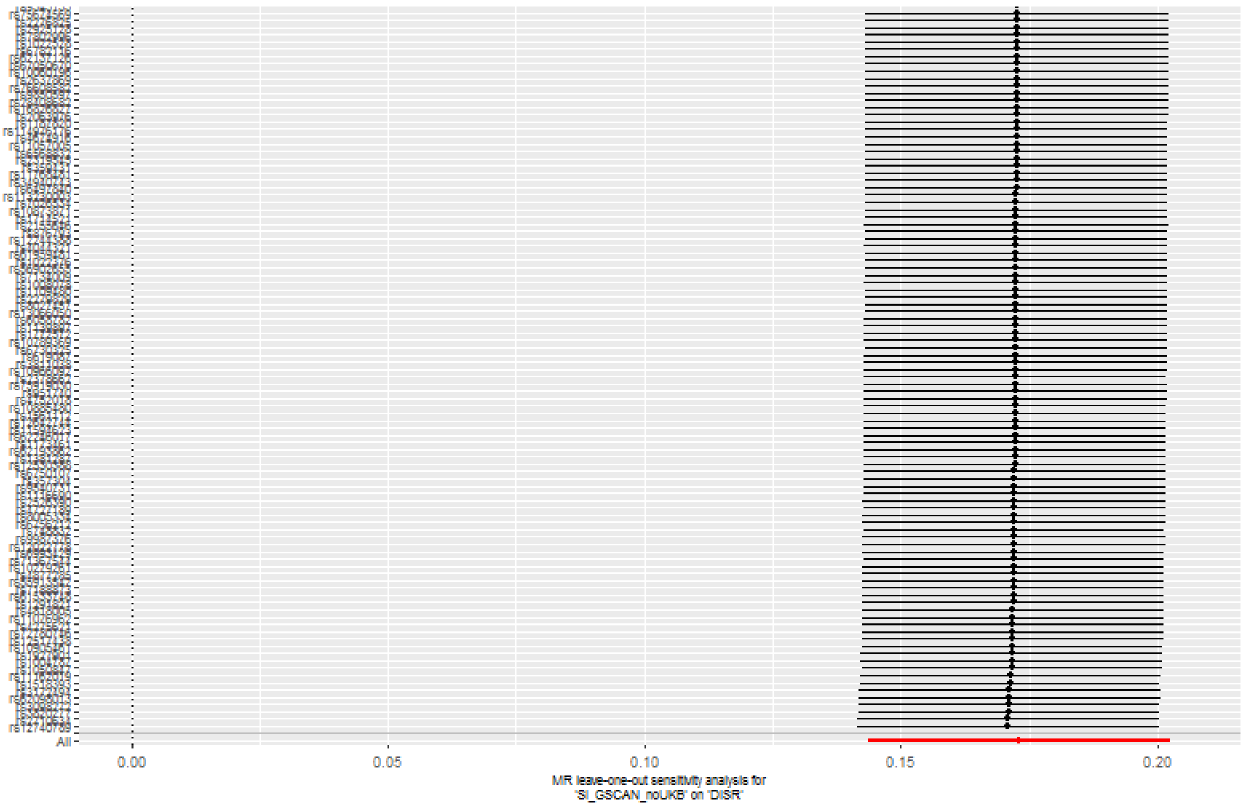

F. From smoking initiation to disruptive behavior.

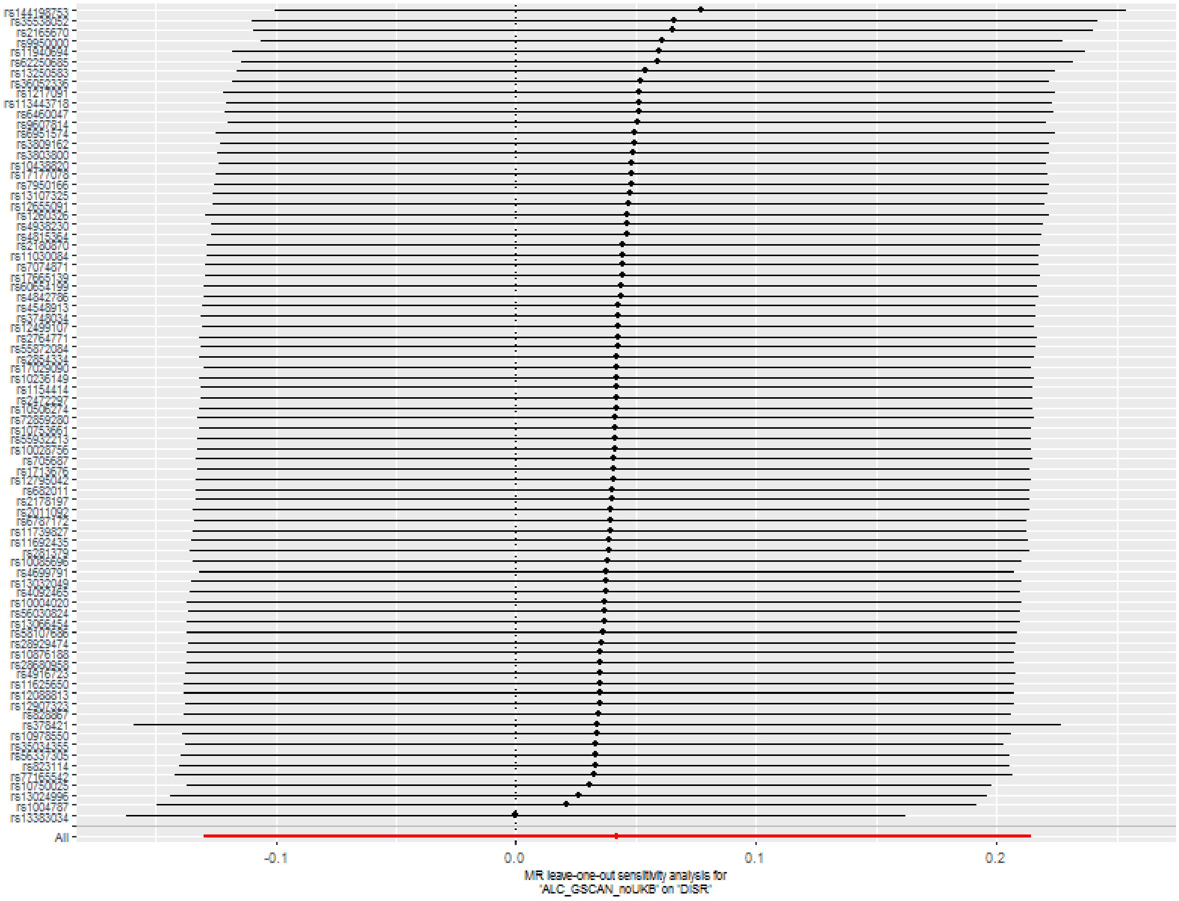

G. From alcoholic drinks per week to disruptive behavior.

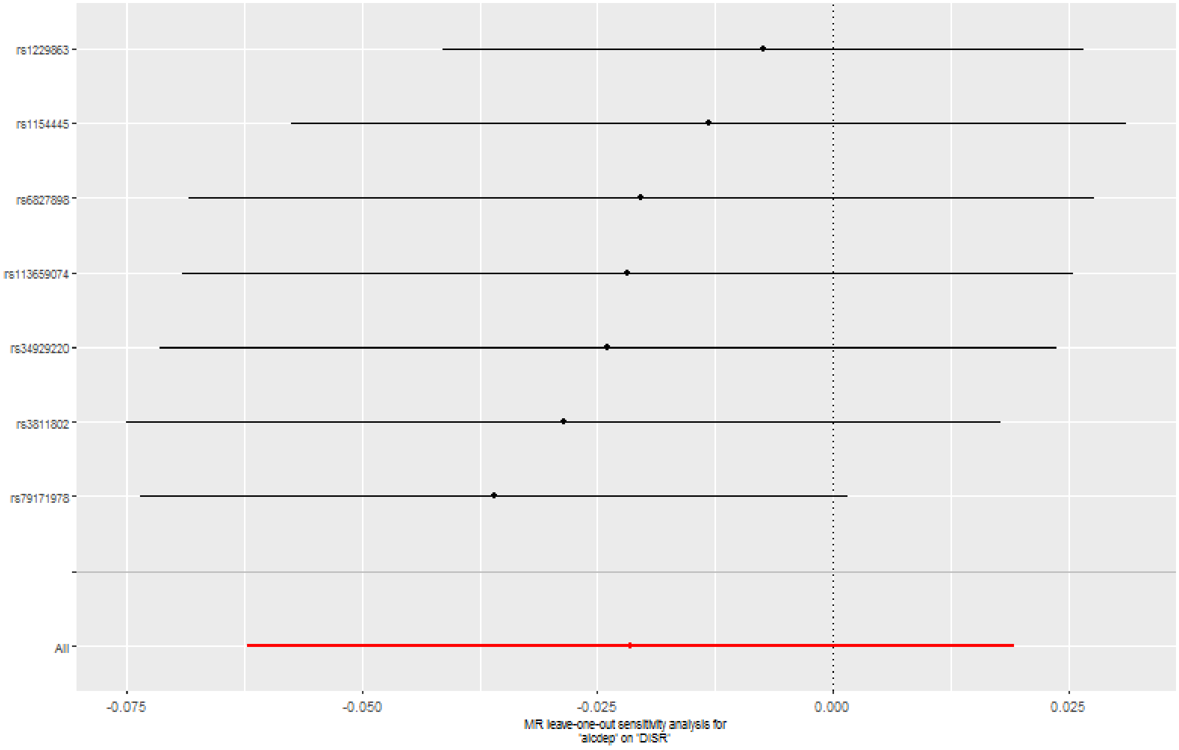

H. From alcohol use disorder, p-value threshold 5E-08, to disruptive behavior.

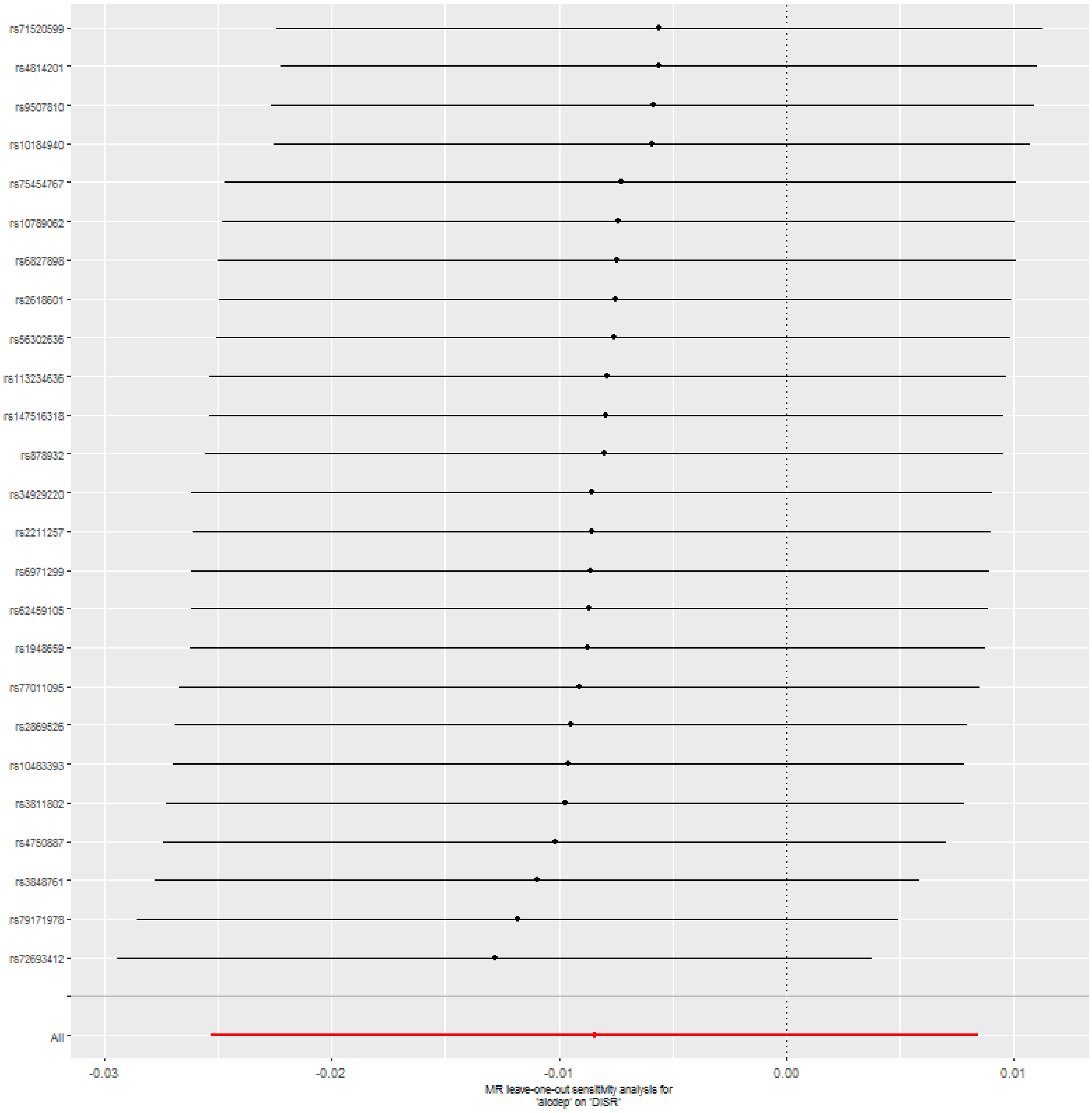

I. From alcohol use disorder, p-value threshold 1E-05, to disruptive behavior.
